# Chronic obstructive pulmonary disease does not impair responses to resistance training

**DOI:** 10.1186/s12967-021-02969-1

**Published:** 2021-07-06

**Authors:** Knut Sindre Mølmen, Daniel Hammarström, Gunnar Slettaløkken Falch, Morten Grundtvig, Lise Koll, Marita Hanestadhaugen, Yusuf Khan, Rafi Ahmad, Bente Malerbakken, Tore Jørgen Rødølen, Roger Lien, Bent R. Rønnestad, Truls Raastad, Stian Ellefsen

**Affiliations:** 1 Section for Health and Exercise Physiology, Inland Norway University of Applied Sciences, P.O. Box 422, 2604 Lillehammer, Norway; 2 Department of Medicine, Innlandet Hospital Trust, Lillehammer, Norway; 3 Department of Pathology, Innlandet Hospital Trust, Lillehammer, Norway; 4 Department of Biotechnology, Inland Norway University of Applied Sciences, Hamar, Norway; 5 Institute of Clinical Medicine, Faculty of Health Sciences, UiT – The Arctic University of Norway, Tromsø, Norway; 6 Lillehammer Hospital for Rheumatic Diseases, Lillehammer, Norway; 7 Innlandet Hospital Trust, Granheim Lung Hospital, Follebu, Norway; 8 Department of Physical Performance, Norwegian School of Sport Sciences, Oslo, Norway; 9 Innlandet Hospital Trust, Lillehammer, Norway

**Keywords:** Anabolic resistance, COPD, Pathophysiology, Skeletal muscle, Strength training, Training load

## Abstract

**Background:**

Subjects with chronic obstructive pulmonary disease (COPD) are prone to accelerated decay of muscle strength and mass with advancing age. This is believed to be driven by disease-inherent systemic pathophysiologies, which are also assumed to drive muscle cells into a state of anabolic resistance, leading to impaired abilities to adapt to resistance exercise training. Currently, this phenomenon remains largely unstudied. In this study, we aimed to investigate the assumed negative effects of COPD for health- and muscle-related responsiveness to resistance training using a healthy control-based translational approach.

**Methods:**

Subjects with COPD (n = 20, GOLD II-III, FEV_1predicted_ 57 ± 11%, age 69 ± 5) and healthy controls (Healthy, n = 58, FEV_1predicted_ 112 ± 16%, age 67 ± 4) conducted identical whole-body resistance training interventions for 13 weeks, consisting of two weekly supervised training sessions. Leg exercises were performed unilaterally, with one leg conducting high-load training (10RM) and the contralateral leg conducting low-load training (30RM). Measurements included muscle strength (n_variables_ = 7), endurance performance (n_variables_ = 6), muscle mass (n_variables_ = 3), muscle quality, muscle biology (*m. vastus lateralis*; muscle fiber characteristics, RNA content including transcriptome) and health variables (body composition, blood). For core outcome domains, weighted combined factors were calculated from the range of singular assessments.

**Results:**

COPD displayed well-known pathophysiologies at baseline, including elevated levels of systemic low-grade inflammation ([c-reactive protein]_serum_), reduced muscle mass and functionality, and muscle biological aberrancies. Despite this, resistance training led to improved lower-limb muscle strength (15 ± 8%), muscle mass (7 ± 5%), muscle quality (8 ± 8%) and lower-limb/whole-body endurance performance (26 ± 12%/8 ± 9%) in COPD, resembling or exceeding responses in Healthy, measured in both relative and numeric change terms. Within the COPD cluster, lower FEV_1predicted_ was associated with larger numeric and relative increases in muscle mass and superior relative improvements in maximal muscle strength. This was accompanied by similar changes in hallmarks of muscle biology such as rRNA-content↑, muscle fiber cross-sectional area↑, type IIX proportions↓, and changes in mRNA transcriptomics. Neither of the core outcome domains were differentially affected by resistance training load.

**Conclusions:**

COPD showed hitherto largely unrecognized responsiveness to resistance training, rejecting the notion of disease-related impairments and rather advocating such training as a potent measure to relieve pathophysiologies.

*Trial registration:* ClinicalTrials.gov ID: NCT02598830. Registered November 6th 2015, https://clinicaltrials.gov/ct2/show/NCT02598830

**Supplementary Information:**

The online version contains supplementary material available at 10.1186/s12967-021-02969-1.

## Introduction

Chronic obstructive pulmonary disease (COPD) is associated with impaired cardiorespiratory fitness and decreased skeletal muscle mass and strength [1], leading to reduced levels of daily activity and reduced quality of life [2, 3]. This deterioration is accompanied by systemic co-morbidities such as reduced levels of testosterone [4], vitamin D [5, 6] and oxygen saturation levels [7], and elevated levels of low-grade inflammation [8], which arguably leaves COPD subjects in a state of anabolic resistance [9], resulting in impaired abilities to adapt to exercise training [10–12]. In particular, these pathophysiologies are believed to impair adaptations to resistance training, which represent the most potent intervention for improving muscle functions [13–16] and preventing escalation into late-stage morbidities such as pulmonary cachexia [17]. Despite this general belief, the presence of anabolic resistance in COPD subjects and its consequences for responses to resistance training remain circumstantial. A mere single study has compared functional and biological adaptations to resistance training between COPD and healthy controls (ISRCTN ID: 22764439) [18–20], and as such was limited by a relatively short training intervention (8 weeks), a rather untraditional training protocol with little clinical and practical relevance, and a limited selection of outcome variables. Whereas the study failed to disclose COPD-related impairments in muscle strength and growth responses, it seems premature to dismiss the notion that COPD pathophysiologies may impair training responsiveness [21], and there is clearly need for further study.

The primary aim of the present study was to investigate the assumed negative effects of COPD pathophysiologies on physiological responses to 13 weeks of resistance training, with emphasis on a broad range of muscle functional and biological outcome measures. The secondary aim was to investigate inherent differences between COPD and Healthy, and to investigate the interaction between two different resistance training modalities and training responsiveness (high-load vs. low-load resistance training; 10 vs 30 repetitions maximum, RM).

## Methods

**Fig. 1:**
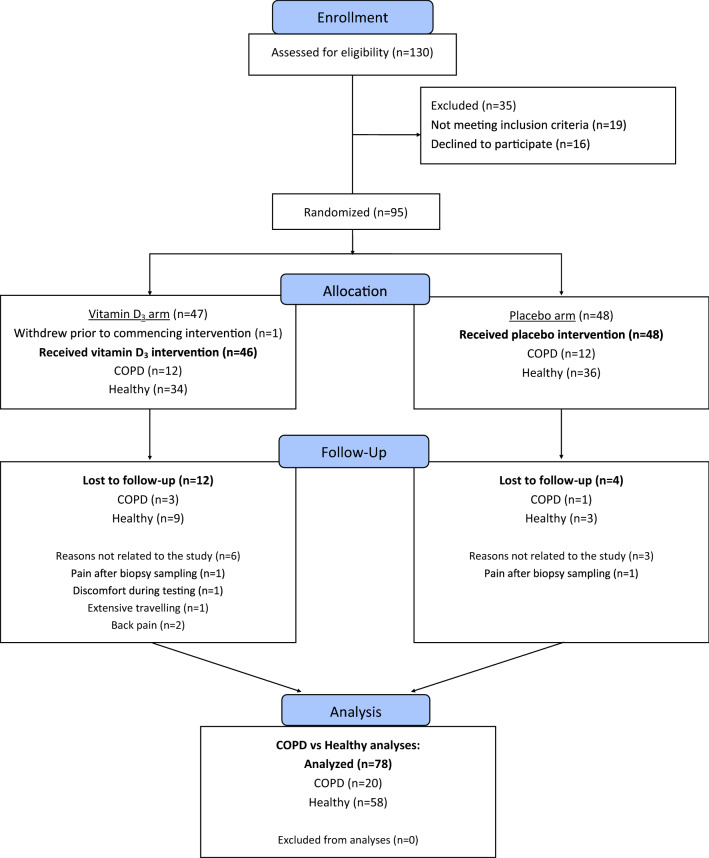
CONSORT flow chart of the study. The study was conducted as a double-blind randomized clinical trial, with the primary aim of investigating the effects of vitamin D_3_ supplementation on resistance training-associated adaptations in a mixed population of older subjects, including both COPD and healthy control subjects (COPD and Healthy, respectively) (ClinicalTrials.gov Identifier: NCT02598830). Vitamin D_3_ supplementation did not affect any primary or secondary outcome, and no conditional effects were observed for COPD vs Healthy in that context [22]. In the present study, the main purpose was to compare the effects of resistance training between COPD and Healthy participants (number of participants completing the study protocol; n COPD = 20; n Healthy = 58)

**Fig. 2:**
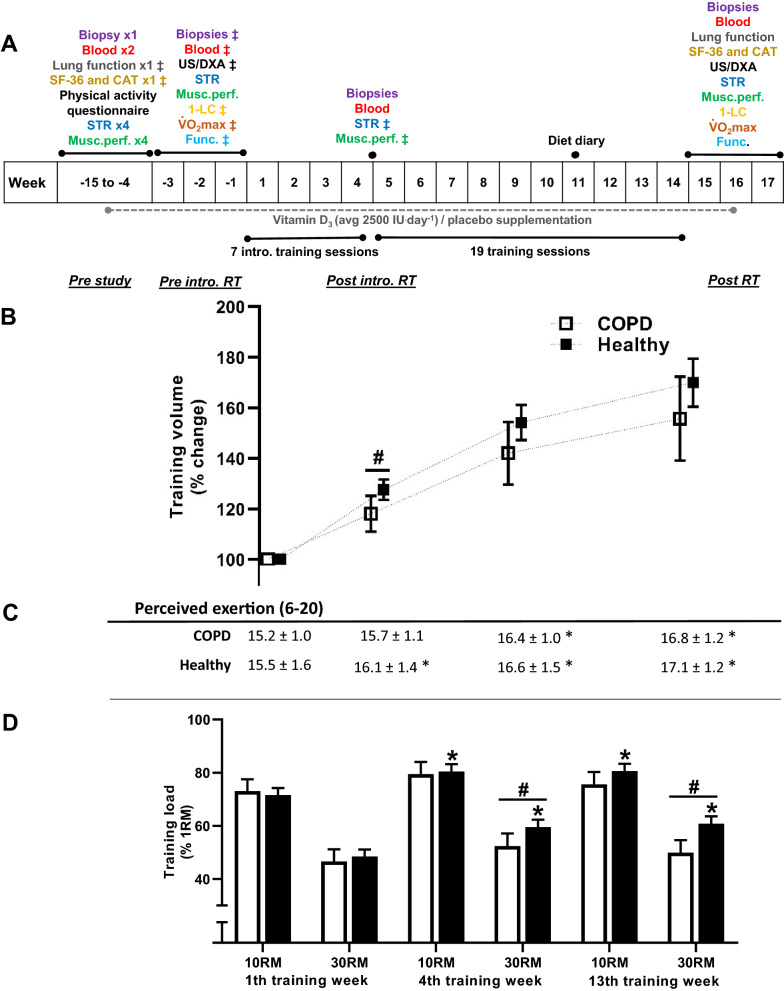
Schematic overview of the study protocol, including its time line (**A**; ^‡^indicates the defined baseline measurement for the specific outcome measure), training volumes during the resistance training (RT) intervention (**B**), perceived exertion (Borg RPE, 6–20) reported after training sessions (**C**), and relative training loads (% of 1RM) during the training period (**D**). Training volume is presented as average increases in per-session for lower-body appendices from the first week of training (kg ^.^ repetitions; high-load (10RM) and low-load (30RM) leg press and knee extension combined). Training loads in numeric values (kg) during the resistance training intervention are provided in Additional file 1: Fig. S1. COPD, participants diagnosed with chronic obstructive pulmonary disease; Healthy, healthy control participants; *statistical different from 1th training week; ^#^statistical difference between COPD and Healthy. Data are presented as means with 95% confidence limits. Methodological notes on retrieval of outcome measures: **i)** Lung function. Spirometry testing was performed following the guidelines from the American Thoracic Society and the European Respiratory Society [72]. Participants with COPD were tested before and after inhalation of two bronchodilators (salbutamol/ipratropiumbromid). **ii)** Muscle strength and performance (STR and Musc. perf). Muscle strength was assessed as one-repetition maximum (1RM) in unilateral knee extension and leg press, bilateral chest press, and handgrip. Muscle performance was defined as the number of repetitions achieved at 50% of pre-study 1RM and was assessed using unilateral knee extension and bilateral chest press. Isokinetic unilateral knee-extension torque was tested at three angular speeds (60°, 120° and 240°^.^ sec^−1^; Humac Norm, CSMi, Stoughton, MA, USA). **iii)** One-legged cycling and bicycling performance (1-LC and VO_2_max). Participants conducted one-legged cycling tests (Excalibur Sport, Lode BV, Groningen, the Netherlands) to assess O_2_-costs and mechanical efficiency [73] during submaximal cycling, and maximal one-legged oxygen consumption (V̇O_2_max) and maximal workload. Maximal two-legged cycling V̇O_2_max and workload were tested on a separate day. Oxygen consumption was measured using the JAEGER Oxycon Pro™ system (Carefusion GmbH, Höchberg, Germany). **iv)** Functional performance (Func.). Functional tests were conducted as the maximal number of sit-to-stands during one minute (seat height: 45 cm) and as the number of steps onto a 20 cm step box during 6 min. **v)** Health-related quality of life (SF-36 and CAT). All participants completed the Short Form (36-item) Health Survey (SF-36). COPD participants also completed the COPD Assessment Test (CAT) questionnaire. **vi)** Muscle thickness and body mass composition (US/DXA). Muscle thickness of *m. vastus lateralis* and *m. rectus femoris* were measured using B-mode ultrasonography (SmartUs EXT-1 M, Telemed, Vilnius, Lithuania). Body mass composition was measured using dual-energy X-ray absorptiometry (DXA; Lunar Prodigy, GE Healthcare, Madison, WI, USA). At pre study, all participants completed a questionnaire regarding regular weekly activity habits. The results (time spent for different activities) were translated into energy expenditure (kcals^.^week^−1^) during activities using number of metabolic equivalents provided in Jetté et al. [74]. During week 11, all participants conducted a dietary registration, in which they logged their dietary intake for three days, including one weekend day

For in-depth description of study protocols and methods, including description of a placebo-controlled vitamin D_3_ supplementation protocol (randomized clinical trial), see Figs. 1, 2 and clinicaltrial.gov (ClinicalTrials.gov Identifier: NCT02598830). The study was designed and scaled to allow elucidation of the effects of vitamin D_3_ supplementation for adaptations to resistance training, as well as to compare training responsiveness between COPD and Healthy. The vitamin D_3_ perspective is covered in detail elsewhere [22].

### Study ethics and participants

The study was approved by the Regional Committee for Medical and Health Research Ethics (reference no. 2013/1094), preregistered at clinicaltrials.gov (NCT02598830), and conducted according to the Declaration of Helsinki. All participants were informed about the potential risks and discomforts associated with the study and gave their informed consent prior to study enrolment.

Persons with either medical diagnosis of stable COPD (GOLD grade II-III [23], predicted forced expiratory volume in first second (FEV_1_) between 80%-30%, FEV_1_/forced vital capacity (FVC) < 70% after reversibility testing, n = 24, age 70 ± 5) or normal lung function (n = 70, age 67 ± 5) received the study intervention. For study flow chart, see Fig. 1. For baseline characteristics of the participants completing the study, see Table 1.

**Table 1:** Characteristics of the participants completing the study

	COPD	Healthy	Sex-adjusted estimated difference	
			COPD – Healthy (95% CI)	*P*-value
General				
Participants, completing (no. ♂/♀) / dropouts† (no.)	20 (12/8) / 2	58 (21/37) / 2	–	–
Age (years)	69 ± 5 (range, 60–79)	67 ± 4 (range, 57–78)	2 (0, 5)	0.049*
Height (cm)	171 (10)	170 (10)	−3 (−6, 0)	0.056
Body mass (kg)	73 (18)	76 (16)	−7 (−14, 0)	0.061
Body mass index (kg^.^ m^2^)	25 (5)	26 (5)	−2 (−4, 1)	0.237
Pack-years (no.)	30 (16)	6 (10)	23 (17, 29)	< 0.001*
GOLD grade (no. of grade II/III)	15/5	–	–	–
COPD Assessment Test™ score (0–40)	16.6 (6.8)	–	–	–
Self-reported conception of health (0–10)	4.9 (1.2)	6.7 (1.6)	− 1.7 (− 2.5, − 0.7)	0.001*
Physical activity level				
Household work (kcals^.^week^−1^)	1754 (2062)	1866 (2201)	− 164 (− 1322, 995)	0.779
Recreational activities (kcals^.^week^−1^)	2512 (2619)	2654 (1841)	188 (− 862, 1237)	0.723
Total activity (kcals^.^week^−1^)	4266 (4036)	4520 (2837)	24 (− 1657, 1704)	0.978
Pulmonary function				
FVC (L)	3.2 (0.9)	3.6 (0.9)	− 0.7 (− 1.0, − 0.4)	< 0.001*
FVC (% predicted)	97 (19)	112 (16)	− 13 (− 22, − 4)	0.003*
FEV_1_ (L^.^ sec^−1^)	1.5 (0.4)	2.7 (0.7)	− 1.4 (− 1.6, − 1.2)	< 0.001*
FEV_1_ (% predicted)	57 (11)	104 (16)	− 47 (− 55, − 39)	< 0.001*
FEV_1_/FVC (%)	47 (8)	75 (6)	− 28 (− 31, − 24)	< 0.001*
PEF (L^.^ sec^−1^)	5.0 (1.6)	7.7 (2.1)	− 3.4 (− 4.1, − 2.7)	< 0.001*
Pulmonary medication				
B_2_-agonists (no.)	17/20	–	–	–
Muscarinic agonists (no.)	15/20	–	–	–
Medication containing both b_2_-agonist and glucocorticoid (no.)	10/20	–	–	–
Body composition				
Total lean mass (kg)	♂, 53 (4); ♀, 36 (6)	♂, 60 (5); ♀, 41 (4)	− 6 (− 9, − 4)	< 0.001*
Whole-body bone mineral density (g^.^ cm^2^)	♂, 1.2 (0.1); ♀, 1.0 (0.2)	♂, 1.3 (0.1); ♀, 1.1 (0.1)	− 0.1 (− 0.2, − 0.0)	0.007*
Total fat mass (kg)	♂, 26 (10); ♀, 27 (15)	♂, 26 (9); ♀, 25 (10)	1 (− 5, 7)	0.703
Visceral fat (kg)	♂, 1.9 (1.3); ♀, 1.0 (0.7)	♂, 1.7 (1.0); ♀, 0.8 (0.7)	0.2 (− 0.3, 0.7)	0.412
Lower−-body muscle strength				
1RM leg press (kg)	♂, 121 (35); ♀, 82 (21)	♂, 152 (27); ♀, 124 (25)	− 36 (− 47, − 26)	< 0.001*
1RM knee extension (kg)	♂, 21 (4); ♀, 11 (4)	♂, 31 (5); ♀, 16 (3)	− 7 (− 9, − 5)	< 0.001*
Peak torque knee extension 60° ^.^ sec^−1^ (Nm)	♂, 127 (34); ♀, 80 (25)	♂, 160 32); ♀, 101 (16)	− 27 (− 36, − 17)	< 0.001*
Peak torque knee extension 180° ^.^ sec^−1^ (Nm)	♂, 83 (25); ♀, 47 (17)	♂, 102 (23); ♀, 62 (11)	− 19 (− 28, − 9)	< 0.001*
Peak torque knee extension 240° ^.^ sec^−1^ (Nm)	♂, 68 (20); ♀, 38 (14)	♂, 84 (20); ♀, 50 (9)	− 15 (− 20, − 9)	< 0.001*
Lower-body muscle strength factor (AU)	♂, 0.5 (0.1); ♀, 0.3 (0.1)	♂, 0.6 (0.1); ♀, 0.4 (0.1)	− 0.1 (− 0.2, − 0.1)	< 0.001*
Lower-body muscle mass measures				
Leg lean mass (kg)	♂, 18 (2); ♀, 12 (3)	♂, 20 (2); ♀, 14 (2)	− 3 (− 4, − 2)	< 0.001*
*M. vastus* lateralis thickness (mm)	♂, 20 (3); ♀, 18 (5)	♂, 22 (3); ♀, 20 (3)	− 2 (− 3, − 1)	0.002*
*M. rectus* femoris thickness (mm)	♂, 13 (4); ♀, 10 (3)	♂, 16 (4); ♀, 15 (4)	− 4 (− 5, − 2)	< 0.001*
Lower-body muscle mass factor (AU)	♂, 0.6 (0.1); ♀, 0.5 (0.1)	♂, 0.7 (0.1); ♀, 0.6 (0.1)	− 0.1 (− 0.2, − 0.1)	< 0.001*
Endurance measures				
Maximal power output one-legged cycling (W)	♂, 73 (13); ♀, 48 (17)	♂, 148 (28); ♀, 108 (21)	− 67 (− 77, − 58)	< 0.001*
Maximal power output two-legged cycling (W)	♂, 118 (38); ♀, 75 (32)	♂, 252 (48); ♀, 167 (32)	− 113 (− 134, − 92)	< 0.001*
Maximal oxygen consumption (mL O_2_^.^ kg^−1.^ min^−1^)	♂, 20 (5); ♀, 16 (5)	♂, 35 (7); ♀, 28 (6)	− 14 (− 18, − 10)	< 0.001*
6 min step test (maximal number of steps)	♂, 123 (35); ♀, 115 (44)	♂, 208 (41); ♀, 196 (38)	− 83 (− 105, − 61)	< 0.001*
1 min sit-to-stand test (maximal number)	♂, 21 (5); ♀, 21 (6)	♂, 30 (5); ♀, 29 (5)	− 9 (− 12, − 6)	< 0.001*
n_repetitions_ at 50% of 1RM knee extension_pre study_	♂, 19 (5); ♀, 17 (5)	♂, 23 (6); ♀, 20 (7)	− 4 (− 6, − 1)	0.005*
One-legged endurance performance factor (AU)	♂, 0.2 (0.0); ♀, 0.2 (0.0)	♂, 0.4 (0.1); ♀, 0.3 (0.1)	− 0.2 (− 0.2, − 0.1)	< 0.001*
Whole-body endurance performance factor (AU)	♂, 0.4 (0.1); ♀, 0.3 (0.1)	♂, 0.7 (0.1); ♀, 0.6 (0.1)	− 0.3 (− 0.3, − 0.2)	< 0.001*

COPD, participants diagnosed with chronic obstructive pulmonary disease; Healthy, healthy control participants; ♂, males; ♀, females; †, dropouts during the training period; *study clusters are significantly different from each other (*p* < 0.05); GOLD, Global Initiative for Chronic Obstructive Lung Disease; pack-years, (number of cigarettes smoked per day/20) × number of years smoked; FVC, forced vital capacity; FEV_1_, forced expiratory volume in one second; PEF, peak expiratory flow; 1RM, one repetition maximum; Nm, newton-meter; AU, arbitrary units. Data mainly presented as mean (SD), and sex-adjusted estimated mean differences between study clusters (95% CI). For core outcome domains, i.e. lower-body muscle strength, lower-body muscle mass, one-legged endurance performance and whole-body endurance performance, factors were calculated. Briefly, each factor was calculated using multiple singular outcome measures, where each of these variables were normalized to the participant with the highest value recorded during the study, resulting in individual scores ≤ 1. Thereafter, outcome domain factors were calculated as the mean of the normalized values for each variable for each subject (see Additional file 1: Table S1 for complete overview over calculations and composition of each factor)

### Study conduct

COPD and Healthy conducted identical 13-week resistance training protocols, consisting of two weekly full-body training sessions (Fig. 2) with primary focus on leg exercises. The leg exercises, i.e. leg press, knee extension and knee flexion, were performed unilaterally in that consecutive order, with one of the legs of each participant being randomly assigned to perform three sets of 10RM (high-load) and the contralateral leg to perform three sets of 30RM (low-load). For each exercise, all three sets for one leg were conducted before the other leg was exercised. This unilateral training protocol served two purposes: i) to circumvent issues relating to conduction of training with two-legged exercises in COPD [24] and ii) to investigate the relative efficacy of two different training modalities (10RM vs 30RM). Exercises and sets were separated by ~ 2 min of rest, with individual adjustments being made whenever participants needed a longer rest period. All sessions were supervised by qualified personnel and lasted for ~ 60 min. The effectiveness of the training intervention was assessed as a wide range of outcome measures (Fig. 2), including multiple assessments of endurance performance, muscle strength and mass, measures of work economy/efficiency, and collection of blood and *vastus lateralis* biopsies (both legs) (Fig. 2).

### Blood and muscle measurements

Prior to collection of blood and muscle biopsies, participants were instructed to attend an overnight fast and to avoid heavy physical activity for the last 48 h. Blood samples were analyzed for serum concentrations of hormones, lipids, and markers of iron metabolism and tissue damage, as previously described [22]. Muscle biopsies were analyzed for muscle fiber type proportions, myonuclei content, muscle fiber cross-sectional area (CSA), and rRNA and mRNA content (total RNA, rRNA subspecies, myosin heavy chain isoforms I, IIA and IIX, and whole-genome transcriptome), as previously described [22, 25, 26]. Transcriptome analysis was restricted to a subset of participants (COPD, n = 19 (n prior to resistance training, 19; n after 3 ½ week of training, 17; n post resistance training, 19); Healthy, n = 34 for all time points), selected based on quality of total RNA samples (RNA Quality Indicator > 7.0, avg 9.0 ± 0.5), with participants with COPD and participants with complete sets of muscle biopsies being prioritized.

### Data analyses and statistics

Analyses were conducted per-protocol, due to the translational approach of the study. For continuous variables, linear mixed-effects models were used to examine differences between COPD and Healthy, both at baseline and as responses to resistance training. For the latter, relative and numeric changes from baseline were defined as dependent variables, with COPD/Healthy being defined as the fixed effect. Effects of sex were implemented into the models. Analyses included evaluation of interaction effects with training load (repeated measures/observations from high- and low-load training legs were added to the model for unilateral outcome measures) and sex. Time effects were examined using mixed modelling, with the dependent variable and time points being defined as repeated measures/observations. To describe the relationship between COPD severity and training responses, simple linear regression analyses of core outcome domains change scores and predicted FEV_1_ were performed.

For non-continuous variables, generalized linear mixed-effects models (GLMMs) were used (binomial GLMMs, immunohistochemical fiber type proportion analyses; negative binomial GLMMs, rRNA/mRNA content in quantitative real-time polymerase chain reaction (qPCR) and transcriptome analyses). For transcriptome analyses, gene counts were modelled using the total library size as a fixed effect [27], together with sex and study conditions (time points and COPD/Healthy). Models were iteratively fitted using glmmTMB [28]. Genes were regarded as differentially expressed when the numeric log_2_ fold-change/difference were greater than 0.5 and the adjusted *p*-value (false discovery rate adjusted *per* model coefficient) was below 5% [25]. Moreover, enrichment analyses were performed on Hallmark, Kyoto encyclopedia of Genes and Genomes (KEGG) and Gene Ontology gene sets, using two approaches. First, a non-parametric rank test was performed based on gene-specific minimum significant differences. Second, a gene set enrichment analysis (GSEA) was performed to quantify directional regulation of the gene set. GSEA was performed using the fgsea package [29]. Consensus results (i.e. when both the non-directional rank test and the directional GSEA turned out significant) were interpreted as having greater biological meaning, while Hallmark was interpreted as contributing with the most meaningful stand-alone interpretation, as it reduces the analytical noise by taking into account genes that overlap between gene sets [30]. All gene sets were retrieved using the molecular signature database (version 7.1.) [31]. Overview of gene enrichment analyses with exact *p*-values are presented in Additional file 1: Table S3. A repository containing all transcriptome data and scripts used for transcriptome and enrichment analyses are available at https://github.com/dhammarstrom/rnaseq-copd.

For all immunohistochemical variables, statistical models were weighted for numbers of counted fibers *per* biopsy. This was done to account for the reduced reliability accompanying fewer observations/fibers [22].

To achieve reliable assessment of core outcome domains, and thus to lower the risk of statistical errors, combined factors were calculated for outcome measures relating to *lower-body muscle strength* (composed of values from the variables 1RM knee extension and leg press (I), and peak torque for knee extension at 60, 180 and 240°/sec (II)), *lower-body muscle mass* (leg lean mass (I) and *vastus lateralis* and *rectus femoris* thickness (II)), *one-legged endurance performance* (maximal workload achieved during one-legged cycling (I) and number of repetitions at 50% of 1RM knee extension at pre-study (II)) and *whole-body endurance performance* (maximal workload achieved during bicycling (I), maximal number of steps achieved in a 6-min test (II), and maximal number of sit-to-stands in a 1-min test (III)), as previously described [22]. During factor calculation, each of the underlying variables were normalized to the participant with the highest value recorded during the RCT, resulting in individual scores ≤ 1. Thereafter, outcome domain factors were calculated as the mean of the normalized values for each variable for each participant. For details, see Additional file 1: Table S1.

In all mixed-effects models, a single random effect was used, giving each participant an individual intercept. Statistical significance was set to *p* < 0.05. In both text and figures, data are presented as adjusted, marginal means, with or without 95% confidence intervals, unless otherwise stated. Statistical analyses were performed using SPSS Statistics package version 24 (IBM, Chicago, IL, USA) (statistical models with continuous variables, as well as immunohistochemical fiber type proportions) and R software [32] (statistical analyses of rRNA/mRNA content). Figures were made using Prism Software (GraphPad 8, San Diego, CA, USA) and R software [32].

## Results and discussion

### Baseline characteristics: COPD vs Healthy

#### Exercise capacity, body composition and muscle and blood biology

At baseline (prior to onset of training), COPD displayed impaired exercise capacity compared to Healthy, as expected from previous studies [3, 18, 20, 33]. This was evident as impaired whole-body performance (range: − 41 − 54%, Table 1), and lower-body unilateral muscle strength and endurance performance (− 17 − 30%, Table 1), presumably reflecting the cardiorespiratory and muscular limitations inherent to the condition [21], and likely being decoupled from levels of habitual physical activity, as no difference was observed between study clusters prior to onset of the study (Table 1). Alongside the reduced exercise capacity, COPD had less lean body mass than Healthy (− 13%, Table 1), with 45% of COPD showing signs of sarcopenia, as defined by Baumgartner et al*.* [34]. In the legs, this was manifested as -16% reductions in leg-specific lean mass and − 9/− 24% smaller *vastus lateralis/rectus femoris* thicknesses (Table 1), offering potential explanations for the impaired maximal leg muscle strength. Of note, for markers of muscle mass the difference between study clusters was likely related to traits inherent to the COPD condition rather than to the small age difference between COPD and Healthy (− 2 years; Table 1), as the magnitude of the difference would have implied an annual loss of ~ 2.6 kg lean mass *per* year in the COPD cluster, deviating substantially from the expected loss in this age group (~ 0.5 kg *per* year) [35].

**Fig. 3:**
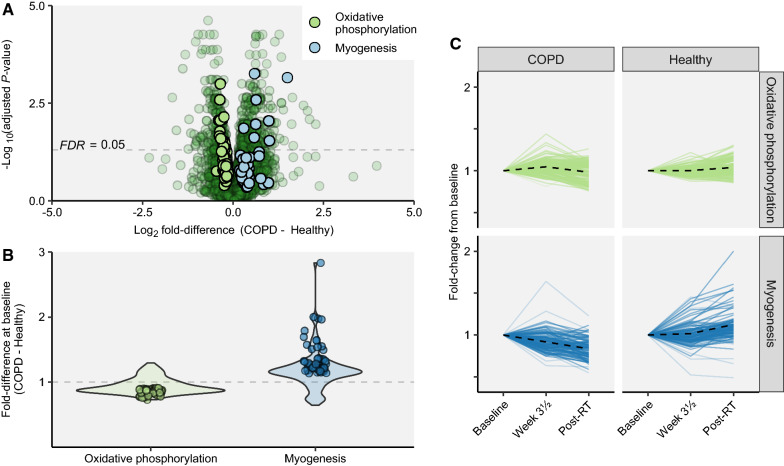
Whole-genome transcriptome analyses of *m. vastus lateralis* in COPD and Healthy (COPD, n = 19; Healthy, n = 34). At baseline, numerous genes were differentially expressed between COPD and Healthy. In **A**, differences in gene expression between COPD and Healthy are presented with leading edge genes (i.e. genes that contributes to the enrichment score) from two gene sets identified as differentially expressed between COPD and Healthy from gene enrichment analyses (*oxidative phosphorylation* and *myogenesis*; see Table 3). In **B**, average fold differences (COPD-Healthy) of genes contributing to baseline differences in *oxidative phosphorylation* and *myogenesis* gene sets are shown as individual data points, and violin plots shows the distribution of all leading edge genes from each gene set. **C** displays the average development of each gene set over time, where the dotted line indicates the mean fold change of all genes contributing to the differential change over time between COPD and Healthy. COPD displayed larger increases in expression of genes relating to *oxidative phosphorylation* after 3½ weeks of training, and more pronounced decreases in genes associated with *myogenesis* to after the training intervention (Post-RT; see Table 3). FDR, false discovery rate-adjusted *p*-value

For muscle biological variables, the COPD cluster showed lowered proportions of type I fibers and greater proportions of type IIA and IIX muscle fibers in *vastus lateralis* compared to Healthy (32/23% vs 13/9%, respectively), corroborating with previous studies [36, 37]. For type I fibers, COPD showed larger CSA (12%, Table 2) and larger myonuclear domain (CSA *per* myonuclei; 20%, Table 2), with no such differences being observed for type II fibers. This contrasts previous studies, who have reported smaller or similar CSA in type I fibers in COPD compared to Healthy [33, 38, 39], and may point to a compensatory mechanism for the likely loss of motor units in COPD subjects [40], whereby reduced quantities of muscle fibers are compensated for by increased sizes of remaining fibers, as previously reported in rodents [41]. These observed differences in muscle fiber characteristics were accompanied by differences in RNA expression. Although COPD and Healthy showed similar levels of total RNA and rRNA expression *per* amount of muscle tissue at baseline (Table 2), COPD displayed distinct whole-genome transcriptome profiles, with 227 genes being differentially expressed compared to Healthy (151↑ and 76↓; Fig. 3A and Additional file 1: Table S2). Hallmark enrichment analysis revealed lower expression of genes involved in *oxidative phosphorylation* (consensus, i.e. agreement between GSEA and rank-based analyses), corroborating with the lower type I proportion, as well as greater expression of genes involved in regulation of *myogenesis* (Rank) (Fig. 3A, B, Table 3; confirmed by gene ontology analysis, Additional file 1: Table S3), which may be related to the pathophysiological elevation of protein turnover in COPD [42, 43].

**Table 2:** Baseline characteristics of *m. vastus lateralis* for COPD and Healthy

	COPD	Healthy	Sex-adjusted estimated difference	
			COPD – Healthy (95% CI)	*P*-value
Cross-sectional area (µm^2^)				
Type I	4614 (1088)	3720 (951)	449 (70, 827)	0.020*
Type II	3639 (1235)	3059 (1121)	182 (− 118, 482)	0.232
Myonuclei per fiber				
Type I	2.2 (0.9)	2.1 (0.9)	− 0.1 (− 0.4, 0.2)	0.357
Type II	2.1 (0.7)	1.9 (0.7)	− 0.1 (− 0.3, 0.2)	0.504
Myonuclear domain (cross sectional area/nuclei per fiber)				
Type I	2292 (585)	1928 (1030)	360 (107, 613)	0.006*
Type II	1775 (529)	1740 (1049)	− 62 (− 316, 191)	0.628
Fiber type proportion (%)				
Type I	52 (15)	65 (14)	− 16 (− 24, − 9)	< 0.001*
Type IIA	32 (12)	23 (11)	10 (4, 16)	0.001*
Type IIX	13 (7)	9 (6)	5 (1, 9)	0.007*
Type IIA/IIX	3 (2)	2 (2)	0.7 (− 0.4, 1.9)	0.159
Total RNA (ng / ml)	477 (103)	504 (106)	− 20 (-59, 18)	0.302

COPD, participants diagnosed with chronic obstructive pulmonary disease; Healthy, healthy control participants. Data presented as mean (SD), and sex-adjusted estimated mean differences between study clusters (95% CI). Alpha level at *p* < 0.05

**Table 3:** Comparison of Hallmark gene sets identified in whole-genome transcriptome data between COPD (n = 19) and Healthy (n = 34), assessed at baseline and as resistance training-associated changes

Comparison	Gene set	Significance category*	Set size^†^	Rank *P*-value^‡^	% MSD > 0^§^	GSEA *P*-value^||^	NES	LE**	Log_2_ fold difference in LE (95% CI)
Baseline: COPD vs Healthy	Oxidative phosphorylation	Consensus	190 (200)	0.007	36.8%	< 0.001	− 2.10	70 (94.3%)	− 0.24 (− 0.45, − 0.13)
	Myogenesis	Rank	163 (200)	< 0.001	33.7%	0.417	1.21	45 (75.6%)	0.46 (0.19, 1.5)
3½ weeks of training: ΔCOPD vs ΔHealthy	Allograft rejection	GSEA	115 (200)	0.956	7.8%	0.014	1.71	20 (35%)	0.39 (0.13, 0.76)
	Oxidative phosphorylation	GSEA	190 (200)	0.999	1.1%	0.009	1.69	83 (2.4%)	0.11 (0.05, 0.39)
	Pancreas beta cells	GSEA	15 (40)	0.969	6.7%	0.028	1.71	3 (33.3%)	0.35 (0.08, 0.54)
Post− RT (13 weeks of training): ΔCOPD vs ΔHealthy	Myogenesis	Consensus	163 (200)	< 0.001	42.3%	< 0.001	− 1.52	68 (85.3%)	− 0.5 (− 1.13, − 0.26)

*Consensus significance indicates agreement between directional (GSEA) and non-directional (Rank) hypothesis test of overrepresentation (see methods for details). ^†^Indicates number of identified genes in the gene set and total number of genes in the gene set in parentheses. ^‡^Rank-based enrichment test, based on minimum significant difference (MSD), identifies gene sets that are overrepresented among top-ranked genes without a directional hypothesis. ^§^ Fraction of genes in gene set with unadjusted 95% CI not spanning zero, i.e. MSD > 0. ^||^ Gene-set enrichment analysis (GSEA) tests for overrepresentation among top and bottom genes based on Log_2_ fold differences or changes × -log_10_(*P*-values) in comparing differences at baseline or changes from baseline between COPD and Healthy. A positive normalized enrichment score (NES) indicate gene set with higher expression in COPD than Healthy; negative NES indicate gene set with lower expression at respective time-points. ** Number of genes in leading edge (LE, genes that contributes to the enrichment score) with the fraction of leading edge genes with unadjusted 95% CI not spanning zero. *∆* change score

For blood variables, the COPD cluster showed elevated levels of low-grade inflammation compared to Healthy, measured as levels of c-reactive protein prior to the study (5.0 vs 1.6 mg^.^L^−1^, *p* = 0.001, data not shown; baseline (i.e. prior to resistance training), 5.0 vs 1.6 mg^.^L^−1^, *p* = 0.053, Table 4), as expected from previous studies [8]. For other characteristics, including hormonal status in blood (e.g. testosterone), no differences were observed between COPD and Healthy (Table 4).

**Table 4:** Effects of the training intervention on body composition and blood variables in COPD and Healthy, assessed as changes from baseline to after completion of the study (per study cluster) and as differential changes between study clusters

	COPD			Healthy			∆ COPD vs∆ Healthy (*P* value)
	Baseline	Post RT	Time effect* (P* < 0.05)	Baseline	Post RT	Time effect (*P* < 0.05)	
Dual-energy x-ray absorptiometry							
Whole-body bone mineral density (g ^.^ cm^2^)	1.13 (0.21)	1.13 (0.21)	No	1.15 (0.16)	1.14 (0.15)	No	0.119
Total lean mass (kg)	46.7 (9.9)	47.6 (10.2)	Yes ↑	48.1 (10.0)	48.6 (10.0)	Yes ↑	0.395
Appendicular lean mass (kg)	20.3 (5.3)	20.9 (5.5)	Yes ↑	21.6 (5.0)	21.9 (5.0)	Yes ↑	0.166
Total fat mass (kg)	26.4 (11.7)	26.3 (11.5)	No	25.3 (9.3)	24.4 (9.2)	Yes ↓	0.068
Visceral fat (kg)	1.59 (1.18)	1.56 (1.21)	No	1.12 (0.98)	1.01 (0.81)	Yes ↓	0.138
Inflammation							
C-reactive protein (mg ^.^ L^−1^)	3.4 (5.0)	3.6 (4.0)	No	1.7 (2.5)	1.8 (3.5)	No	0.934
Hormones							
Cortisol (nmol ^.^ L^−1^)	307 (130)*	310 (109)	No	369 (88)	372 (99)	No	0.861
Growth hormone (µg ^.^ L^−1^)	1.4 (2.8)	1.4 (3.1)	No	1.1 (1.7)	1.3 (1.6)	No	0.837
IGF-1 (nmol ^.^ L ^−1^)	15.7 (4.2)	15.0 (4.5)	No	14.4 (3.2)	13.6 (3.1)	Yes ↓	0.977
Testosterone (nmol ^.^ L^−1^)†	11.2 (4.4)	11.4 (4.2)	No	11.9 (3.3)	12.4 (4.2)	No	0.938
Sex-hormone binding globulin (nmol ^.^ L^−1^)	60 (33)	60 (34)	No	60 (22)	60 (21)	No	0.488
Androstenedione (nmol ^.^ L^−1^)	3.3 (2.4)	3.3 (2.4)	No	3.8 (2.7)	3.8 (2.4)	No	0.984
Parathyroid hormone (pmol ^.^ L^−1^)	5.7 (2.6)	6.0 (3.3)	No	5.0 (2.2)	5.2 (1.9)	No	0.870
Lipid profile variables							
Triglycerides (mmol ^.^ L^−1^)	1.2 (0.5)	1.1 (0.5)	No	1.2 (0.5)	1.1 (0.6)	Yes ↓	0.661
HDL (mmol ^.^ L^−1^)	1.6 (0.6)	1.7 (0.5)	No	1.7 (0.5)	1.7 (0.5)	No	0.523
LDL (mmol ^.^ L^−1^)	2.8 (1.0)*	2.8 (1.0)	No	3.4 (1.0)	3.3 (0.8)	No	0.775
Iron biology variables							
Fe^2+^ (µmol ^.^l L^−1^)	18 (7)	18 (6)	No	18 (6)	18 (5)	No	0.410
Transferrin (g ^.^ L^−1^)	2.66 (0.44)*	2.67 (0.45)	No	2.41 (0.27)	2.38 (0.29)	No	0.563
Ferritin (µg ^.^ L^−1^)	113 (92)	90 (81)	Yes ↓	139 (79)	133 (68)	No	0.089
Calcium status							
Calcium (mmol ^.^ L^−1^)	2.4 (0.1)	2.4 (0.1)	No	2.4 (0.1)	2.4 (0.1)	No	0.865
Albumin-corrected calcium (mmol ^.^ L^−1^)	2.3 (0.1)	2.3 (0.1)	No	2.3 (0.1)	2.3 (0.1)	No	0.802
Tissue damage variables							
Aspartate transaminase (units ^.^ L^−1^)	27 (9)	24 (6)	No	26 (21)	26 (7)	No	0.807
Creatine kinase (units ^.^ L^−1^)	112 (69)	123 (71)	No	95 (47)	125 (72)	Yes ↑	0.523

Body composition analyses, n COPD = 19, n Healthy = 48; blood analyses, n COPD = 20, n Healthy = 58. *significant difference between COPD and Healthy at baseline; †only men were included in testosterone analysis; ↓significant decrease from baseline to post RT (after 13 weeks of resistance training); ↑significant increase from baseline to post RT. Alpha level at *p* < 0.05. Data are presented as means (SD)

### The efficacy of the resistance training intervention: COPD vs Healthy

For both COPD and Healthy, the training intervention was associated with low drop-out rates (n = 4, ~ 5%; COPD, n = 2), high adherence to the protocol (COPD, 97%; Healthy, 98%; measured as the average number of training sessions completed), progressive increases in training volume (Fig. 2), and robust increases in muscle strength *per* training session (e.g. 1RM knee extension, 0.9% ^.^ session^−1^/0.8% ^.^ session^−1^, COPD/Healthy; 1RM leg press, 1.4% ^.^ session^−1^/1.3% ^.^ session^−1^). The habitual dietary intake was similar between COPD and Healthy, with protein intake being 1.2 (0.3) and 1.3 (0.4) g ^.^ kg^−1 .^ day^−1^, respectively, complying with current guidelines [44]. The vitamin D_3_ supplementation RCT of the project did not enhance or affect training-associated changes for any of the primary or secondary outcome measures [22].

#### Muscle strength, muscle mass, muscle quality and one-legged endurance performance

**Fig. 4:**
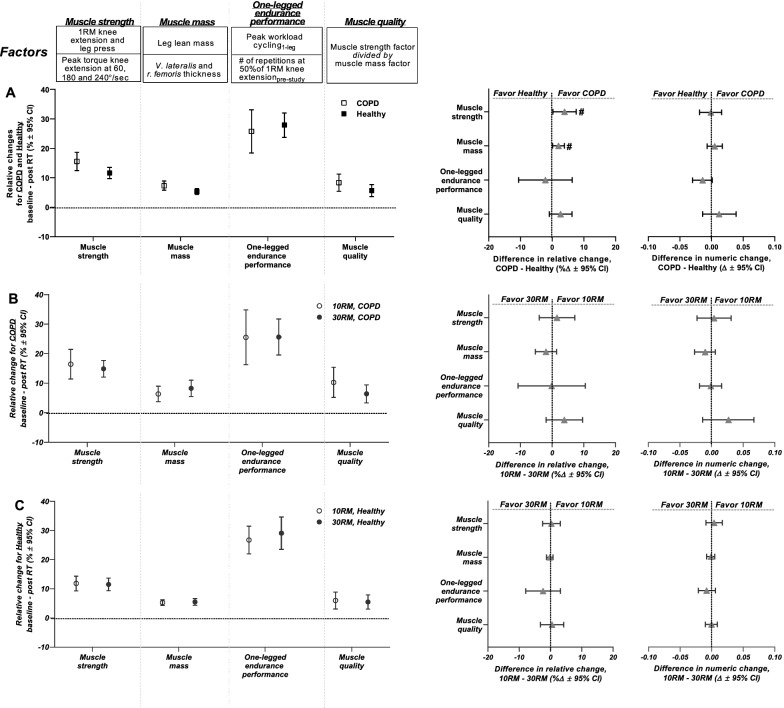
Effects of the resistance training intervention on lower-body muscle strength (COPD, n = 18; Healthy, n = 50), lower-body muscle mass (COPD, n = 19; Healthy, n = 47), one-legged endurance performance (COPD, n = 15; Healthy, n = 49) and lower-body muscle quality (COPD, n = 18; Healthy, n = 38) in COPD and Healthy. Each outcome domain is represented by a combined factor, computed from various performance assessments, as defined in the upper panel of the figure and previously described [22]. **A** presents comparison of overall training effects between COPD and Healthy, measured as relative changes from baseline to after the resistance training intervention (per study cluster; left panel) and as relative and numeric differences in change scores between study clusters (right panels). In these analyses, high- and low-load resistance training (10RM and 30RM, respectively) were combined, warranted by the lack of differences between training load conditions in (B, C). COPD showed greater relative changes in muscle strength and muscle mass than Healthy. **B**, **C** presents comparison of effects of 10RM and 30RM resistance training in COPD (**B**) and Healthy (**C**) (i.e. per study cluster), measured as relative changes from baseline to after the intervention (left panels) and as relative and numeric differences in change scores between load conditions (right panels). ^#^statistically different effects of resistance training between COPD and Healthy. Data are presented as means with 95% confidence limits

Overall, COPD showed larger training-associated increases in lower-body muscle strength and mass compared to Healthy (the two legs/training modalities combined), measured as relative changes in combined factors from baseline (Fig. 4A), with no difference being observed for numeric changes (Fig. 4A). COPD and Healthy showed similarly scaled improvements in muscle quality and one-legged endurance performance (Fig. 4A). Within the COPD cluster, worsening of lung function (i.e. decreasing predicted FEV_1_ values) was associated with larger numeric and relative increases in muscle mass, as well as larger relative improvements in maximal muscle strength, with no such relationship being observed for muscle quality or one-legged endurance performance (Table 5). Neither of the four core outcome domains (muscle strength/mass/quality or one-legged endurance performance) were differentially affected by resistance training load (neither in COPD nor in Healthy), suggesting that 30RM training is an effective alternative to 10RM training in older individuals (Fig. 4B, C). Of note, the comparisons between 10 and 30RM training responses may have been confounded by the so-called *cross-education effect*, whereby training of one limb affects functional and biological characteristics of the contralateral limb. However, the true existence of such cross-education effects remains disputed, and if it does exist, its impact is likely restricted to neuromuscular functionality [45, 46], with no apparent effects on muscle biological measures such as mRNA abundance [45], mitochondrial content [47, 48], capillarization [49], muscle protein synthesis [50] or muscle hypertrophy [51, 52]. In accordance with this, the cross-education effect may have affected measures of muscle strength and one-legged endurance performance in the present study. Importantly, however, several measures were implemented into the study protocol to minimize its impact, including extensive familiarization to training and physical testing (e.g. baseline muscle strength was measured after 3 ½ weeks of introduction to training and was preceded by 3–5 familiarization sessions to muscle strength testing) [22].

**Table 5:** Simple linear regression analyses on the relationship between training response and lung function in COPD participants

Analysis	n	Slope (95% CI)	Intercept (95% CI)	*r*	*P*
Change in muscle strength vs FEV_1predicted_					
% change	18	− 0.3 (− 0.6, 0.0)	34.8 (16.8, 52.9)	− 0.504	0.033
Numeric change	18	− 0.001 (− 0.003, 0.001)	0.121 (0.017, 0.225)	− 0.303	0.222
Change in muscle mass vs FEV_1predicted_					
% change	19	− 0.3 (− 0.4, − 0.1)	21.4 (12.1, 30.7)	− 0.624	0.004
Numeric change	19	− 0.002 (− 0.003, 0.000)	0.127 (0.068, 0.186)	− 0.603	0.006
Change in muscle quality vs FEV_1predicted_					
% change	18	− 0.1 (− 0.4, 0.2)	12.6 (− 4.2, 29.4)	− 0.141	0.577
Numeric change	18	0.000 (− 0.002, 0.002)	0.063 (− 0.060, 0.186)	− 0.038	0.881
Change in one-legged endurance performance vs FEV_1predicted_					
% change	15	0.3 (− 0.4, 1.0)	8.5 (− 32.8, 49.7)	0.249	0.371
Numeric change	15	0.001 (− 0.001, 0.002)	0.006 (− 0.066, 0.079)	0.282	0.308
Change in whole-body endurance performance vs FEV_1predicted_					
% change	17	− 0.2 (− 0.6, 0.3)	17.7 (− 7.8, 43.1)	− 0.211	0.416
Numeric change	17	0.000 (− 0.001, 0.001)	0.023 (− 0.042, 0.089)	0.012	0.963

FEV_1predicted_, predicted forced expiratory volume in first second; *r*, Pearson’s *r*; *P*, *P*-value

Overall, COPD showed marked and hitherto unrecognized responsiveness to resistance training in respect of improvements in muscle strength, muscle mass, muscle quality and one-legged endurance performance, contradicting previous suggestions of a negative impact of co-morbidities such as low cardiorespiratory fitness and chronic low-grade systemic inflammation [8, 24]. Indeed, a more severe COPD diagnosis was associated with larger increases in muscle mass and muscle strength improvements. This observation cannot be readily explained by baseline differences between the COPD participants (e.g. baseline muscle mass vs predicted FEV_1_, *p* = 0.998; baseline muscle strength vs predicted FEV_1_, *p* = 0.646). The marked training responsiveness in COPD was presumably also decoupled from initial differences in physical activity habits between study clusters, as COPD and Healthy showed similar characteristics regarding these measures (Table 1), though some caution is warranted for interpretation of such self-reported recall questionnaire results [53].

#### Cycling and functional performance

**Fig. 5:**
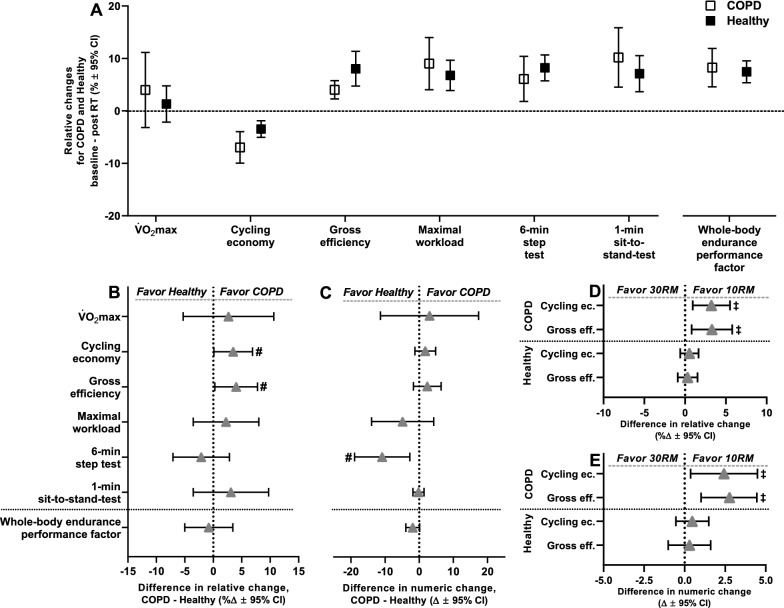
Comparison of the effects of the resistance training intervention on whole-body endurance performance in COPD and Healthy, presented as relative changes from baseline (per study cluster; **A**) and as relative and numeric differences in change scores between study clusters (**B** and **C**, respectively). Endurance measures included maximal oxygen consumption (V̇O_2_max, cl^.^ min^−1^; COPD, n = 15; Healthy, n = 54) and maximal workload (watts; COPD, n = 18; Healthy, n = 55) achieved during two-legged cycling, cycling economy (cl^.^ min^−1^; COPD, n = 15; Healthy, n = 54) and gross efficiency measured during submaximal one-legged cycling, the number of steps achieved during 6-min step test (COPD, n = 18; Healthy, n = 57) and the number of sit-to-stands achieved during a 1-min sit-to-stand test (COPD, n = 19; Healthy, n = 56). COPD showed greater relative improvements in cycling economy and gross efficiency. For these outcome measures, COPD, but not Healthy, displayed benefits of high-load training (10RM) compared to low-load training (30RM) (**D** and **E**). Healthy showed greater numeric improvement in the number of steps achieved during the 6-min step test. COPD and Healthy showed similar relative and numeric training-associated changes in the whole-body endurance performance factor. ^#^statistically different response to resistance training between study clusters. ^‡^statistically different response to 10RM and 30RM resistance training in study cluster. Data are presented as means with 95% confidence limits

COPD and Healthy showed pronounced and similarly scaled training-associated improvements in whole-body endurance performance, measured as changes from baseline, including 6-min step test performance, 1-min sit-to-stand performance and maximal workload achieved during two-legged cycling (Fig. 5). Surprisingly, COPD and Healthy also showed similar changes in performance for these outcome measures in numeric terms, with exception of 6-min step test performance, for which Healthy showed larger improvements (COPD, 6 steps; Healthy, 17 steps; ∆11 steps, *p* = 0.009; Fig. 5), arguably related to the considerable cardiorespiratory demand of this test, leaving COPD with morbidity-specific restraints. Corroborating with this, within the COPD cluster, there was no association between the severity of the COPD diagnosis and resistance training-induced changes in whole-body endurance performance (Table 5). For other performance indices such as cycling economy and gross efficiency, which were measured using a one-legged cycling protocol, COPD showed larger relative improvements compared to Healthy (∆4% (COPD – Healthy) for both cycling economy and gross efficiency, Fig. 5). For these indices of cycling performance, COPD, but not Healthy, displayed benefits of 10RM compared to 30RM training (Fig. 5), corresponding to previously observed effects of heavy resistance training in healthy, young individuals [54].

Together, these observations reiterate on the substantial benefits of resistance training for subjects with COPD, even for performance measures that pose large whole-body metabolic demands, which has previously been suggested to be irresponsive to such training [55]. As such, it seems plausible that the observed improvements in 6-min step test performance, 1-min sit-to-stand performance and two-legged cycling were associated with improvements in work economy/gross efficiency and muscle strength, as neither COPD nor Healthy showed training-associated changes in maximal oxygen consumption (Fig. 5), with improvements in anaerobic capacity being a potential contributor (not measured).

#### Muscle fiber characteristics

**Fig. 6:**
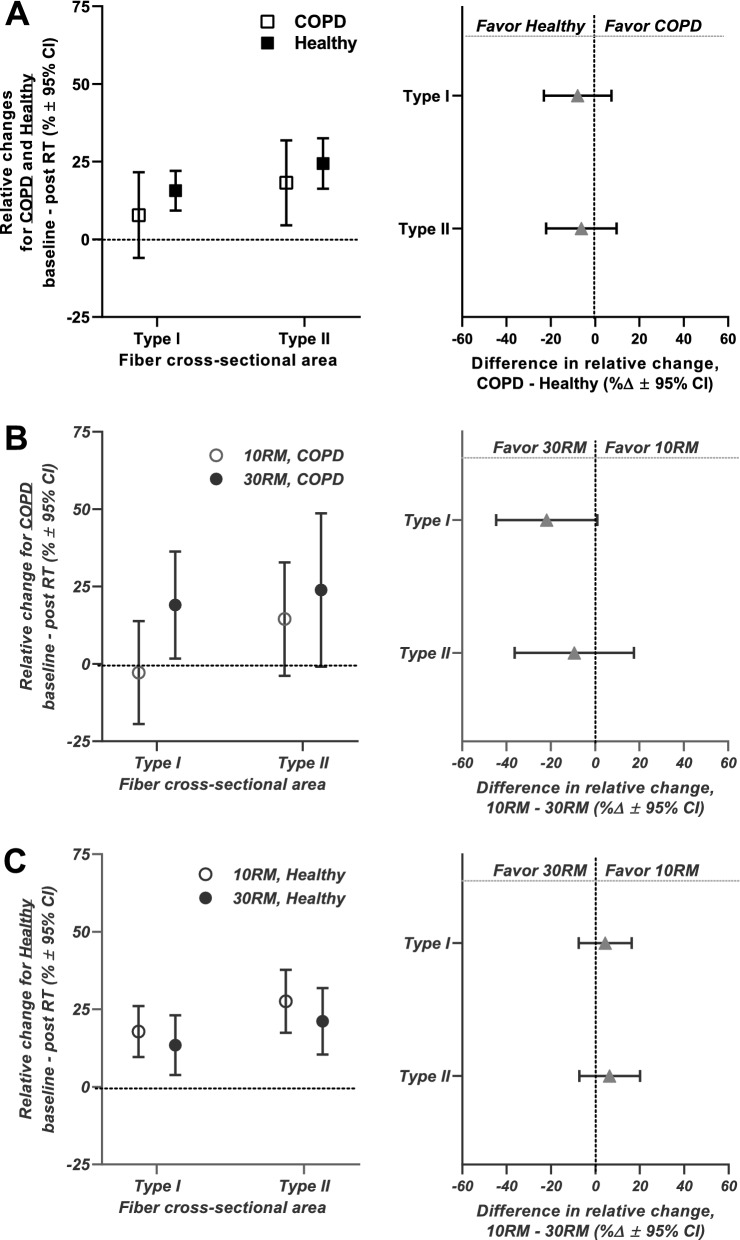
Effects of the resistance training intervention on cross-sectional area of muscle fiber types I and II in *m. vastus lateralis* in COPD (n = 18) and Healthy (n = 55). **A** presents comparison of overall training effects on fiber CSA between COPD and Healthy, measured as relative changes from baseline to after the training intervention (per study cluster; left panel) and as relative differences in change scores between study clusters (right panel). In these analyses, high- and low-load resistance training (10RM and 30RM, respectively) were combined, warranted by the lack of significant differences between training load conditions in (**B**, **C**), though COPD tended to show higher efficacy of 30RM resistance training for changes in fiber type I CSA. **B**, **C** presents comparisons of effects of 10RM and 30RM resistance training on fiber CSA in COPD (**B**) and Healthy (**C**) (i.e. per study cluster), measured as relative changes from baseline to after the training intervention (left panels) and as relative and numeric differences in change scores between load conditions (right panels). Data are presented as means with 95% confidence limits

**Fig. 7:**
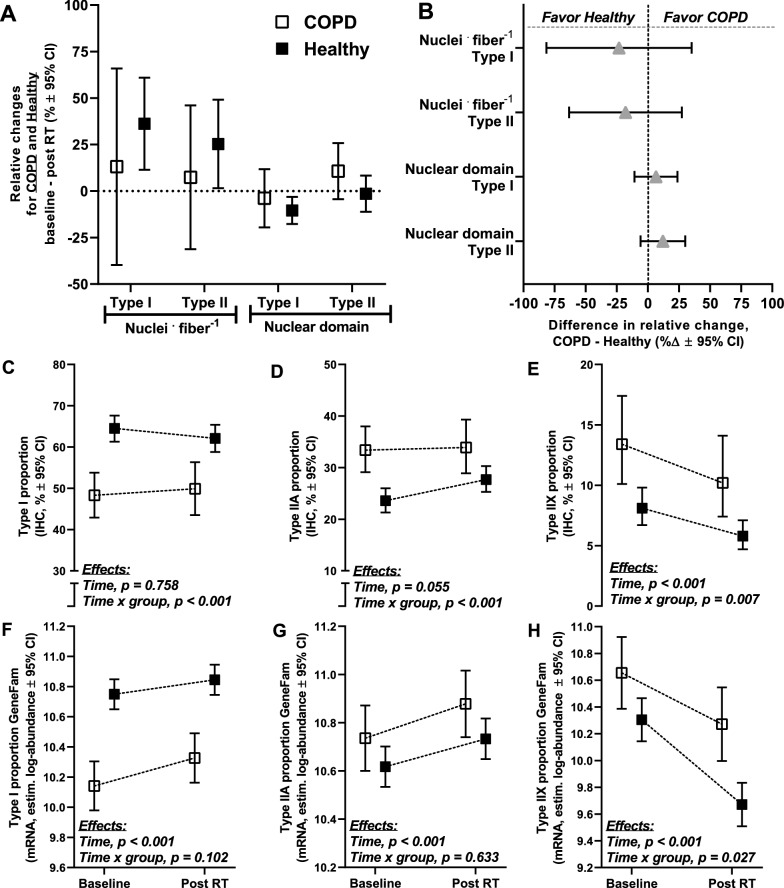
Comparisons of the effects of the resistance training intervention on changes in myonuclei *per* fiber and myonuclei domain in muscle fiber types I and II (**A**, **B**; COPD, n = 11; Healthy, n = 34), and on changes in muscle fiber type proportions in COPD and Healthy, measured using immunohistochemistry (**C**–**E**; COPD, n = 17; Healthy, n = 51)) and qPCR (gene family profiling-normalized myosin heavy chain mRNA expression, **F**–**H**; COPD, n = 19; Healthy, n = 55), as previously described [26, 75]. Myonuclei domain was calculated as mean fiber cross-sectional area divided by myonuclei *per* fiber. For myonuclei *per* fiber and myonuclei domain in muscle fiber types I and II, comparisons are presented as relative changes from baseline to after the training intervention (per study cluster; **A**) and as relative differences in change scores between study clusters (**B**). For muscle fiber type proportions, data are presented as adjusted values at baseline and after the training intervention (Post RT), and results are presented as the effect of the training intervention for the study clusters combined and its interaction with study clusters (**C**–**H**). For myonuclei variables, no training-associated differences were observed between study clusters. Both COPD and Healthy displayed training-associated reductions in proportions of type IIX muscle fibers, measured using both immunohistochemistry and qPCR. Intriguingly, while this reduction was greater in COPD when measured at the protein level (immunohistochemistry), it was greater in Healthy when measured at the mRNA level (qPCR), indicating differentially regulated muscle fiber shifting in COPD and Healthy. Data are presented as means with 95% confidence limits

Whereas COPD and Healthy displayed similar increases in type II fiber CSA in *m. vastus lateralis* in response to resistance training (COPD, 18%; Healthy, 24%; ∆-6%, *p* = 0.438; Fig. 6, upper panel), only Healthy showed increases in type I fiber CSA (16%), with no statistical difference being observed between study clusters. For Healthy, the increase in CSA was accompanied by increased myonuclei ^.^ fiber^−1^ in both fiber types (36%/25% for type I/II; Fig. 7), leading to decreased myonuclear domain size estimates in type I fibers (-10%, Fig. 7). For COPD, no such effects were observed (Fig. 7). Despite the lack of difference between the two study clusters for these variables, the data hints at blunted plasticity of type I muscle fibers in COPD only, potentially relating to their altered biological characteristics at baseline or to blunted myonuclear accretion. Interestingly, in sub-analyses, the blunted type I responses in COPD seemed to be specific to 10RM training, with a tendency towards superior responses to 30RM training (10RM, -3%; 30RM, 19%; ∆22%, *p* = 0.060; Fig. 6, middle panel). Such a phenomenon is supported by previous observations in responses to blood-flow-restricted low-load training [56], which arguably is mimicked by COPD subjects during low-load training, as they display inherent lowering of oxygen saturation in blood.

Both COPD and Healthy displayed training-associated reductions in type IIX muscle fiber proportions (Fig. 7). While this reduction was more pronounced in COPD when measured at the protein level (immunohistochemistry), it was more pronounced in Healthy when measured at the mRNA level, suggesting differential orchestration of muscle fiber shifts between study clusters, possibly relating to their inherently different muscle fiber proportions at baseline.

#### Muscle RNA content

**Fig. 8:**
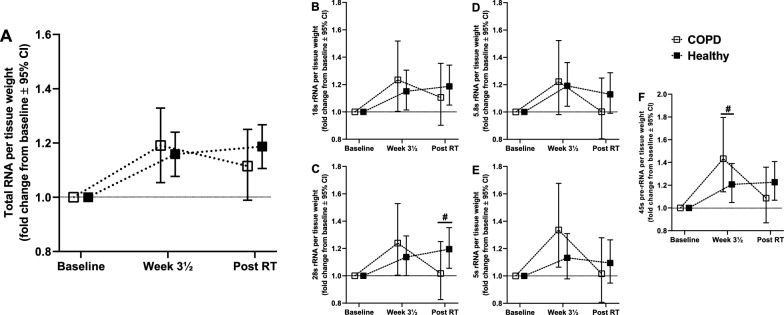
Effects of the resistance training intervention on total RNA content (**A**) and rRNA expression (**B**–**F**) in *m. vastus lateralis* of COPD (n = 19) and Healthy (n = 55). Data are presented as fold changes from baseline to Week 3½ (Post-intro RT; seven training sessions) and to after the training intervention (Post RT; 26 training sessions). Total RNA (**A**), 18s rRNA (**B**), 28s rRNA (**C**), 5.8s rRNA (**D**) 5s rRNA (**E**) and 45s pre-rRNA (**F**) abundances. Total RNA- and qPCR-analyses were assessed as per-amounts of tissue weight, as previously described [22, 26]. ^#^statistical difference in fold change between COPD and Healthy (alpha level, *p* < 0.05). Data are presented as means with 95% confidence limits

In general, COPD and Healthy showed similar increases in ribosomal RNA abundance *per* unit muscle tissue weight, measured as both total RNA and rRNA expression, and measured after both 3½ week (1.19/1.29 and 1.16/1.16 fold increases, total RNA/rRNA abundances) and after finalization of the training intervention (1.12/1.05 and 1.19/1.17 fold increases) (Fig. 8). While these changes in ribosomal RNA content were generally similar between COPD and Healthy, a few noteworthy differences were evident, including a more robust early increase in 45s pre-rRNA abundance in COPD (Fig. 8) and a trend towards reduced changes in response to 13 weeks training in COPD, which led to the absence of time effects for all rRNA species. The early increases in ribosomal content seen in both COPD and Healthy resemble those typically seen after similar interventions in untrained young individuals [26], and may be important for muscle growth capabilities over the entirety of the study period [26, 57], accommodating increases in protein synthesis capacity, thus potentially contributing to the pronounced muscular responses to resistance training seen in both study clusters.

In both COPD and Healthy, resistance training led to marked changes in mRNA transcriptome profiles, with 499 and 312 differentially expressed genes being observed after 3½ and 13 weeks of resistance training, respectively (for general information about transcriptomic responses, see Mølmen et al. [22]). Overall, at the single-gene level, no transcripts showed differential responses to training between the two study clusters, neither at 3½ weeks nor at 13 weeks, despite clear differences in transcriptome profiles at baseline (Fig. 3A and Additional file 1: Table S2). In contrast, enrichment analyses revealed traces of differential changes (Fig. 3C, Table 3 and Additional file 1: TableS 3), with COPD showing more pronounces increases in expression of genes relating to *oxidative phosphorylation* after 3½ weeks (GSEA), and, in particular, more pronounced decreases in genes associated with *myogenesis* after 13 weeks (consensus) (Fig. 3C, Table 3). Interestingly, as these two gene sets represented the most prominent differences between COPD and Healthy at baseline (Fig. 3A, B), and as resistance training led to directional changes that mitigated these differences, training arguably shifted the COPD phenotype in a healthy direction.

#### Blood and health-related outcomes

Overall, COPD and Healthy showed similar training-associated increases in whole-body and appendicular lean mass (Table 4). This was accompanied by increased appendicular skeletal muscle mass index relative to the sex-specific mean of young, healthy adults (COPD, from 84 to 86%; Healthy, from 95 to 97%), suggesting that the intervention was effective for reversing age-related decline in muscle mass. For blood variables such as markers of systemic inflammation and hormone, lipid and iron biology, no noteworthy effects were observed of the intervention, nor were any differential changes observed between COPD and Healthy (Table 4).

#### Lung function

For COPD, the training intervention did not affect any of the lung function variables (Table 6), implying no effects on this core epidemiological trait. This seems reasonable given the irreversible nature of the respiratory impairments of COPD, contradicting the beneficial effects observed in Hoff et al. [14] In contrast, for Healthy, the intervention was associated with reduced FVC and FEV_1_ (− 2.7% and − 1.5%, respectively). Rather than being a consequence of the intervention protocol per se, this may be due to a general age-related decline, as the magnitude of the changes resemble those seen in corresponding age cohorts over a similar time frame [58].

**Table 6:** Effects of the training intervention on lung function in COPD (n = 20) and Healthy (n = 58), assessed as changes from baseline to after completion of the study (per study cluster) and as differential changes between study clusters

	COPD			Healthy			
	Baseline	Post RT	Time effect p < 0.05)	Baseline	Post RT	Time effect (p < 0.05)	∆ COPD vs ∆ healthy (*p*-value)
FVC (L)	3.3 ± 0.9	3.2 ± 0.9	No	3.6 ± 0.9	3.5 ± 0.8	Yes↓	0.189
FEV_1_ (L ^.^ sec^−1^)	1.5 ± 0.4	1.5 ± 0.4	No	2.7 ± 0.7	2.7 ± 0.6	Yes↓	0.243
FEV_1_ (% predicted)	56 ± 11	58 ± 13	No	103 ± 16	103 ± 16	No	0.138
FEV_1_/FVC (%)	47 ± 8	48 ± 10	No	75 ± 6	76 ± 6	No	0.714
PEF (L ^.^ sec^−1^)	5.0 ± 1.6	5.1 ± 1.6	No	7.8 ± 2.1	7.6 ± 2.2	No	0.238

*FVC* forced vital capacity, *FEV*_*1*_ forced expiratory volume in one second, *PEF* peak expiratory flow, *∆* change score, ↓significant decrease from baseline to post RT (after 13 weeks of resistance training). Alpha level at *p* < 0.05. Values are means with standard deviation

#### Health-related quality of life

For COPD, the intervention was associated with marked improvements in several aspects of health-related quality of life (Table 7). These included reduced experience of limitations of physical functioning and improved social function and mental health, with only marginal effects being seen in Healthy. While these changes of course may be directly related to the resistance training intervention, they may also be related to other aspects of the study protocol, such as performing training sessions in a social setting and the close follow-up each participant received from study personnel. As the intervention was conducted without a control group (not receiving the intervention protocol), caution is warranted for interpretation of these data.

**Table 7:** Effects of the training intervention on health-related quality of life in COPD and Healthy, measured using COPD Assessment Test (CAT; COPD-only, n = 20) and the 36-item Short Form Health Survey (SF-36; all participants; n COPD = 20, n Healthy = 57), and assessed as changes from baseline to after completion of the study (per study cluster; CAT and SF-36) and as differential changes between study clusters (SF-36)

	COPD			Healthy			∆ COPD vs ∆ Healthy (*P* value)
	Baseline	Post RT	Time effect *P* < 0.05)	Baseline	Post RT	Time effect (*P* < 0.05)	
COPD assessment Test™ score (0–40)	16.6 ± 6.8	16.4 ± 6.8	No	–	–	–	–
Short Form (36) Health Survey (0–100)							
Physical function*	63 ± 19	67 ± 18	No	90 ± 14	92 ± 12	No	0.321
Role physical*	43 ± 34	59 ± 37	Yes^↑^	87 ± 25	94 ± 18	No	0.226
Bodily pain	71 ± 27	82 ± 19	Yes^↑^	79 ± 21	80 ± 19	No	0.070
General health*	48 ± 20	56 ± 19	No	75 ± 18	80 ± 12	No	0.208
Vitality*	52 ± 16	57 ± 13	No	72 ± 18	78 ± 11	Yes^↑^	0.509
Social function*	74 ± 23	84 ± 16	Yes^↑^	90 ± 18	94 ± 13	No	0.280
Role emotional*	65 ± 39	84 ± 26	Yes^↑^	93 ± 19	96 ± 15	No	0.059
Mental health*	77 ± 13	84 ± 13	Yes^↑^	86 ± 11	89 ± 8	Yes^↑^	0.196

*difference between COPD and Healthy at baseline; ^↑^significant increase from baseline to after the training intervention (Post RT). Alpha level at *p* < 0.05. Values are means with standard deviation

## Concluding remarks

COPD-related pathophysiologies, such as reduced testosterone [4], vitamin D [5] and oxygen saturation levels [7, 59] in blood, and elevated levels of low-grade inflammation [8], are generally believed to drive metabolism into a chronic catabolic state [4, 7, 9]. This has been suggested to lead to impaired responses to lifestyle interventions such as resistance training [7, 60], which are essential measures for preventing and treating disease-related reductions in skeletal muscle mass and strength, counteracting escalation into serious conditions such as pulmonary cachexia [17]. Despite this general belief, the presence of impaired training responsiveness in COPD is not backed by experimental data, and there is limited de facto evidence for such impairments. To date, a mere single study has compared responses between COPD and healthy control subjects [18–20], and as such failing to lend support to the prevailing view, though being limited by a relatively short time span (8 weeks) and a restricted selection of outcome variables. In the present study, we largely disavow the myth of impaired responsiveness to training in COPD, measured as responses to a 13-week whole-body resistance training intervention, conducted using an exhaustive follow-up and testing protocol, which included extensive test–retest validations (for details, see Mølmen et al. [22]). Whereas COPD participants displayed clear and well-known disease-related aberrancies compared to Healthy at baseline, including altered skeletal muscle characteristics and elevated levels of systemic inflammation, they showed similar or superior improvements for virtually every measure of health, performance and biology. Specifically, COPD showed greater relative improvements in core outcome domains such as lower-body muscle strength and mass, and similar relative improvements in muscle quality, one-legged endurance performance and whole-body endurance performance. These similarities were also evident in numeric change terms, suggesting that the improvements seen in COPD was decoupled from the compromised levels at baseline. Indeed, within the COPD cluster, worsening of lung function was associated with larger numeric and relative increases in muscle mass, as well as larger relative improvements in maximal muscle strength. These observations were accompanied by similar alterations in muscle biology, including changes in hallmark traits such as muscle fiber characteristics, rRNA content and transcriptome profiles. Together, these data suggest that COPD-related etiologies and pathophysiologies do not impair responsiveness to resistance training, at least not for skeletal muscle characteristics, and at least not in the enrolled cluster of COPD participants (GOLD grade II-III) and within the time frame of the current study.

During planning of the study protocol, two strategies were implemented to resolve the hypothesized, albeit rejected, negative impact of COPD-specific pathophysiologies for the efficacy of resistance training. *First*, as vitamin D insufficiency is common among COPD subjects [5], and has been suggested to contribute to development of anabolic resistance [61], dietary habits were manipulated to investigate the effects of vitamin D_3_ supplementation. Contrary to our hypothesis, vitamin D_3_ did not enhance responses to resistance training for any of the outcome variables [22]. *Second*, the resistance training protocol was conducted using two different training modalities, 10RM and 30RM resistance training, performed in a contralateral manner. The efficacies of these training modalities were initially hypothesized to be dissimilarly affected by COPD-related pathophysiologies, as they convey muscular adaptations through different signaling cues in the cellular environment (i.e. mechanical tension vs metabolic perturbation) [62], and may thus well be differentially affected by extracellular signaling such as inflammation and oxygen availability. While this hypothesis was rejected for all core outcome domains, with no differences being observed between training modalities and no evidence being found for the presence of impaired training responsiveness, a noteworthy observation was made for muscle fiber-specific traits. Specifically, in COPD, 10RM training was associated with blunted growth of type I muscle fiber CSA, a phenomenon that was not observed for responses to 30RM training, suggesting that 30RM offers benefits for muscle fiber type I hypertrophy. In addition to this, 10RM was associated with greater improvements in cycling economy and gross efficiency in COPD. These observations warrant further study. Of note, the unilateral resistance-training design was arguably supportive for the pronounced resistance-training effects in COPD participants. By reducing cardiorespiratory demand, and thus facilitating higher degrees of muscle activation and muscle mass-specific intensities during exercise compared to conventional two-legged resistance exercise [24] this seems to translate into larger functional improvements for this population [16].

*Study limitations*. Functional and physiological responses to resistance training is not uniform in the human population, and covary with individual characteristics such as genetics, epigenetics and composites of the inner physiological milieu [63–65]. For any research project that aim to understand the aetiology of such training, the interpretation of outcome data is thus a complicated task, which is further complicated by our present crude understanding of the interplay between the characteristics in question and their associated response patterns. While these limitations need to be acknowledged also in the current analyses, their presence underlines the importance of making study-design decisions that contribute to increase the ecological validity of the research project. As an example, in the present study, the advent of contralateral training protocols arguably increased the resolution of 10RM vs 30RM comparisons by removing genetic variability as a source of variation, albeit even this perspective may have been affected by additional complications, such as the previously discussed cross-education effect. Furthermore, in any study, it is prudent to monitor, and ideally also account for, exogenous factors that may have impacted the physiological milieu, and therefore also training responses. In the present study, these included surveillance of lifestyle characteristics such as habitual dietary intake, activities of daily living and intake of pulmonary medication. For habitual patterns of dietary intake and activities of daily living, we observed no difference between study clusters (COPD vs Healthy), though it should be acknowledged that the collection of these data were performed only once during the study, and as such were performed using diary/questionnaire, making them prone to validity issues and warranting caution upon their interpretation [53, 66]. For pulmonary medication, the COPD and Healthy clusters deviated from each other for disease-related reasons, with 19 out of 20 COPD participants reporting intake of either beta_2_-agonists, muscarinic agonists, or drugs containing a mixture of beta_2_-agonists and glucocorticoids, as detailed in Table 1. These drugs are known to affect muscle biology and functionality in humans [67–70], and as such may have influenced the outcome of the study. However, the reported medication status of the COPD participants corresponds to what is normal for COPD subjects in general [71], and as such reflects the population intended to be studied.

In conclusion, 13-week resistance training program was well-tolerated by subjects with COPD and led to pronounced improvements for a range of health and muscle functional and biological variables, resembling or exceeding those seen in Healthy, with some outcome measures even showing indices of more beneficial adaptations in COPD participants with a more severe diagnosis. COPD was thus not associated with impaired responsiveness to resistance exercise training, which rather posed a potent measure to relieve disease-related pathophysiologies.

## Supplementary Information

**Additional file 1. **Additional tables and figure.

## Data Availability

A repository containing all transcriptome data and scripts used for transcriptome and enrichment analyses are available at https://github.com/dhammarstrom/rnaseq-copd. For other outcome measures, data are available from the corresponding author on reasonable request.

## Abbreviations

COPD: Chronic obstructive pulmonary disease
RM: Repetition(s) maximum
FEV_1_: Forced expiratory volume in one second
FVC: Forced vital capacity
CSA: Cross-sectional area
GSEA: Gene set enrichment analysis
