# Supplementary material for: Chronic obstructive pulmonary disease does not impair responses to resistance training
Source: J Transl Med. 2021 Jul 6;19:292. doi: 10.1186/s12967-021-02969-1 (PMC8261934; doi:10.1186/s12967-021-02969-1)
Supplement: Supplementary file 1 — Additional file 1. Additional tables and figure. [file 12967_2021_2969_MOESM1_ESM.docx]

**Chronic Obstructive Lung Disease Does Not Impair Responses to Resistance Training**

Knut S. Mølmen; Daniel Hammarström; Gunnar S. Falch; Morten Grundtvig; Lise Koll; Marita Hanestadhaugen; Yusuf Khan; Rafi Ahmad; Bente Malerbakken; Tore J. Rødølen; Roger Lien; Bent R. Rønnestad; Truls Raastad; Stian Ellefsen

Supplementary Material

**Supplementary Figure 1.** Training loads during the resistance training intervention for COPD and Healthy.

**
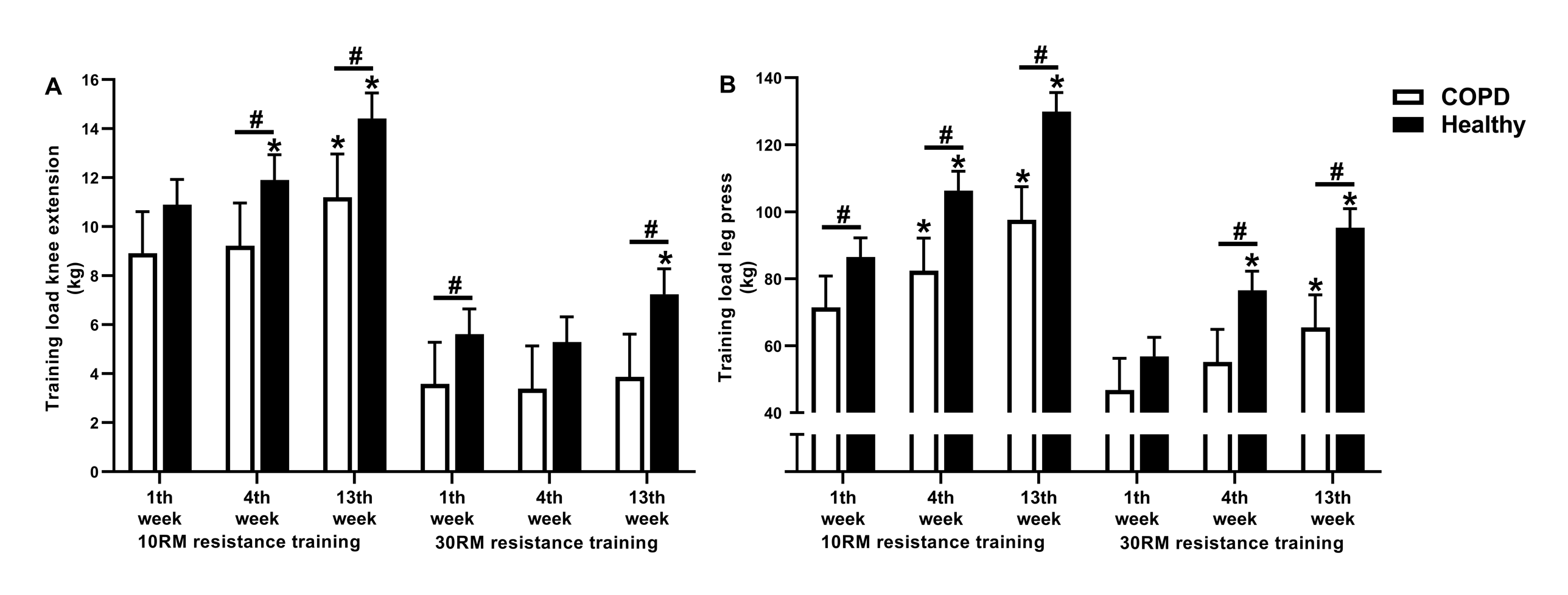
**

COPD, participants diagnosed with chronic obstructive pulmonary disease; Healthy, healthy control participants; *, statistical different from 1th training week; #, statistical difference between COPD and Healthy. Data are presented as means with 95% confidence limits.

**Supplementary Table 1.** Computed factors for the core outcome domains lower-body muscle mass, lower-body muscle strength, one-legged endurance performance and whole-body endurance performance*.* Each factor consists of multiple singular outcome measures. First, for each outcome measure, each subject’s value (pre and post) was normalized to the highest recorded value during the study conduct, thus providing values <1. Then, for each subject, an ultimate factor was computed for lower-body muscle mass, lower-body muscle strength, one-legged endurance performance and whole-body endurance performance, respectively, calculated as the mean of normalized values for the various variables included.

| ***Lower-body muscle mass factor*** | | |  |  |  |  |  |  |  |  |
| --- | --- | --- | --- | --- | --- | --- | --- | --- | --- | --- |
|  | ***Included variables*** | ***Explanation*** | ***Baseline (avg ± SD)*** | ***Post intervention (avg ± SD)*** | ***Estimate, change (95% CI)*** | ***Main effect of time (p value)*** | ***Correlation with factor at baseline, r value (p value)*** | ***Correlation for change score with change score for factor, r value (p value)*** | ***Eigenvalue*** | ***% variance explained*** |
|  | 1. Muscle thickness | The combined measure of muscle thickness of *vastus lateralis* and *rectus femoris* | 0.60 (0.10) | 0.66 (0.11) | 0.06 (0.05, 0.07) | <0.001 | 0.83 (<0.001) | 0.84 (<0.001) | - | - |
|  | 1. Leg lean mass | Lean mass in the legs | 0.64 (0.15) | 0.65 (0.15) | 0.01 (0.01, 0.02) | <0.001 | 0.92 (<0.001) | 0.63 (<0.001) | - | - |
|  | **Lower-body muscle mass factor** | **-** | **0.63 (0.11)** | **0.66 (0.12)** | **0.04 (0.03, 0.04)** | **<0.001** | **-** | **-** | **1.10** | **55** |
|  |  |  |  |  |  |  |  |  |  |  |
|  |  |  |  |  |  |  |  |  |  |  |
| ***Lower-body muscle strength factor*** | | |  |  |  |  |  |  |  |  |
|  | ***Included variables*** | ***Explanation*** | ***Baseline (avg ± SD)*** | ***Post intervention (avg ± SD)*** | ***Estimate, change (95% CI)*** | ***Main effect of time (p value)*** | ***Correlation with factor at baseline, r value (p value)*** | ***Correlation for change score with change score for factor, r value (p value)*** | ***Eigenvalue*** | ***% variance explained*** |
|  | 1. Leg muscle strength | The combined measure of 1RM knee extension and leg press | 0.44 (0.14) | 0.52 (0.15) | 0.08 (0.07, 0.09) | <0.001 | 0.95 (<0.001) | 0.78 (<0.001) | - | - |
|  | 1. Leg muscle torque | The combined measure of torque (Nm) achieved during knee extension at 60°, 180° and 240°/sec | 0.48 (0.16) | 0.51 (0.17) | 0.03 (0.02, 0.04) | <0.001 | 0.97 (<0.001) | 0.73 (<0.001) | - | - |
|  | **Lower-body muscle strength factor** | **-** | **0.46 (0.14)** | **0.52 (0.15)** | **0.06 (0.05, 0.06)** | **<0.001** | **-** | **-** | **1.13** | **57** |
|  |  |  |  |  |  |  |  |  |  |  |
|  |  |  |  |  |  |  |  |  |  |  |
| ***One-legged endurance performance factor*** | | |  |  |  |  |  |  |  |  |
|  | ***Included variables*** | ***Explanation*** | ***Baseline (avg ± SD)*** | ***Post intervention (avg ± SD)*** | ***Estimate, change (95% CI)*** | ***Main effect of time (p value)*** | ***Correlation with factor at baseline, r value (p value)*** | ***Correlation for change score with change score for factor, r value (p value)*** | ***Eigenvalue*** | ***% variance explained*** |
|  | 1. Muscle performance | Number of repetitions at 50% of 1RM knee extension | 0.13 (0.03) | 0.22 (0.09) | 0.09 (0.08, 0.10) | <0.001 | 0.60 (<0.001) | 0.92 (<0.001) |  |  |
|  | 1. Maximal power output | Maximal power output achieved during one-legged cycling | 0.49 (0.17) | 0.52 (0.17) | 0.04 (0.03, 0.04) | <0.001 | 0.99 (<0.001) | 0.29 (<0.001) |  |  |
|  | **One-legged endurance performance factor** | **-** | **0.31 (0.09)** | **0.37 (0.11)** | **0.06 (0.06, 0.07)** | **<0.001** |  |  | **1.11** | **56** |
|  |  |  |  |  |  |  |  |  |  |  |
| ***Whole-body endurance performance factor*** | | |  |  |  |  |  |  |  |  |
|  | ***Included variables*** | ***Explanation*** | ***Baseline (avg ± SD)*** | ***Post intervention (avg ± SD)*** | ***Estimate, change (95% CI)*** | ***Main effect of time (p value)*** | ***Correlation with factor at baseline, r value (p value)*** | ***Correlation for change score with change score for factor, r value (p value)*** | ***Eigenvalue*** | ***% variance explained*** |
|  | Maximal power output | Maximal power output achieved during bicycling | 0.44 (0.17) | 0.47 (0.18) | 0.03 (0.02, 0.04) | <0.001 | 0.87 (<0.001) | 0.54 (<0.001) | - | - |
|  | 6-min step test | Number of steps achieved during a 6-min test | 0.59 (0.17) | 0.63 (0.19) | 0.04 (0.03, 0.06) | <0.001 | 0.96 (<0.001) | 0.64 (<0.001) | - | - |
|  | 1-min sit-to-stand test | Number of sit-to-stands achieved during a 1-min test | 0.58 (0.13) | 0.62 (0.14) | 0.04 (0.03, 0.06) | <0.001 | 0.86 (<0.001) | 0.76 (<0.001) | - | - |
|  | **Whole-body endurance performance factor** | - | 0.54 (0.14) | 0.58 (0.15) | 0.04 (0.03, 0.05) | **<0.001** | - | - | **1.29** | **43** |

**Supplementary Table 2.** Genes identified as differentially expressed at baseline between COPD and Healthy in genome-wide transcriptome analyses (RNA-seq). RNA-seq analyses were performed as previously described.^E1,4^

| **Ensembl gene ID** | **Gene Symbol** | **Log fold-change** | **SE** | ***Z*-value** | ***P*-value** | **Adjusted *P*-value^a^** |  |  |  |  |  |  |  |
| --- | --- | --- | --- | --- | --- | --- | --- | --- | --- | --- | --- | --- | --- |
| ENSG00000146416 | AIG1 | -0.48 | 0.08 | -6.025 | 1.69e-09 | 2.56e-05 |  |  |  |  |  |  |  |
| ENSG00000112796 | ENPP5 | -0.57 | 0.10 | -5.556 | 2.75e-08 | 5.81e-05 |  |  |  |  |  |  |  |
| ENSG00000137942 | FNBP1L | -0.37 | 0.07 | -5.537 | 3.08e-08 | 5.81e-05 |  |  |  |  |  |  |  |
| ENSG00000143507 | DUSP10 | 0.44 | 0.08 | 5.612 | 2.00e-08 | 5.81e-05 |  |  |  |  |  |  |  |
| ENSG00000146477 | SLC22A3 | 0.88 | 0.16 | 5.555 | 2.78e-08 | 5.81e-05 |  |  |  |  |  |  |  |
| ENSG00000152782 | PANK1 | -0.44 | 0.08 | -5.601 | 2.14e-08 | 5.81e-05 |  |  |  |  |  |  |  |
| ENSG00000189067 | LITAF | 0.56 | 0.10 | 5.585 | 2.34e-08 | 5.81e-05 |  |  |  |  |  |  |  |
| ENSG00000205678 | TECRL | -0.67 | 0.12 | -5.620 | 1.91e-08 | 5.81e-05 |  |  |  |  |  |  |  |
| ENSG00000102007 | PLP2 | 0.50 | 0.09 | 5.495 | 3.91e-08 | 5.90e-05 |  |  |  |  |  |  |  |
| ENSG00000133816 | MICAL2 | 0.44 | 0.08 | 5.478 | 4.31e-08 | 5.91e-05 |  |  |  |  |  |  |  |
|  | MICALCL | 0.44 | 0.08 | 5.478 | 4.31e-08 | 5.91e-05 |  |  |  |  |  |  |  |
| ENSG00000120658 | ENOX1 | 0.80 | 0.15 | 5.397 | 6.78e-08 | 8.16e-05 |  |  |  |  |  |  |  |
| ENSG00000150722 | PPP1R1C | -0.71 | 0.13 | -5.391 | 7.02e-08 | 8.16e-05 |  |  |  |  |  |  |  |
| ENSG00000113448 | PDE4D | 0.42 | 0.08 | 5.355 | 8.55e-08 | 9.22e-05 |  |  |  |  |  |  |  |
| ENSG00000048052 | HDAC9 | -0.59 | 0.11 | -5.242 | 1.59e-07 | 1.26e-04 |  |  |  |  |  |  |  |
| ENSG00000105835 | NAMPT | -0.38 | 0.07 | -5.253 | 1.50e-07 | 1.26e-04 |  |  |  |  |  |  |  |
| ENSG00000136040 | PLXNC1 | -0.52 | 0.10 | -5.251 | 1.51e-07 | 1.26e-04 |  |  |  |  |  |  |  |
| ENSG00000073910 | FRY | -0.43 | 0.08 | -5.225 | 1.74e-07 | 1.31e-04 |  |  |  |  |  |  |  |
| ENSG00000151746 | BICD1 | -0.61 | 0.12 | -5.172 | 2.31e-07 | 1.65e-04 |  |  |  |  |  |  |  |
| ENSG00000267296 | CEBPA-DT | 0.56 | 0.11 | 5.165 | 2.40e-07 | 1.65e-04 |  |  |  |  |  |  |  |
| ENSG00000225549 | Not mapped^d^ | -0.92 | 0.18 | -5.146 | 2.66e-07 | 1.73e-04 |  |  |  |  |  |  |  |
| ENSG00000198729 | PPP1R14C | 0.44 | 0.09 | 5.126 | 2.96e-07 | 1.79e-04 |  |  |  |  |  |  |  |
| ENSG00000237301 | Not mapped^d^ | 0.92 | 0.18 | 5.095 | 3.48e-07 | 1.95e-04 |  |  |  |  |  |  |  |
| ENSG00000091879 | ANGPT2 | 0.65 | 0.13 | 4.990 | 6.04e-07 | 2.95e-04 |  |  |  |  |  |  |  |
| ENSG00000151276 | MAGI1 | -0.36 | 0.07 | -4.994 | 5.90e-07 | 2.95e-04 |  |  |  |  |  |  |  |
| ENSG00000196152 | ZNF79 | 0.43 | 0.09 | 4.989 | 6.06e-07 | 2.95e-04 |  |  |  |  |  |  |  |
| ENSG00000183625 | CCR3 | -0.96 | 0.20 | -4.927 | 8.37e-07 | 3.83e-04 |  |  |  |  |  |  |  |
| ENSG00000140416 | TPM1 | 0.46 | 0.09 | 4.871 | 1.11e-06 | 4.78e-04 |  |  |  |  |  |  |  |
| ENSG00000130595 | TNNT3 | 0.41 | 0.08 | 4.856 | 1.20e-06 | 5.02e-04 |  |  |  |  |  |  |  |
| ENSG00000186352 | ANKRD37 | 0.59 | 0.12 | 4.849 | 1.24e-06 | 5.07e-04 |  |  |  |  |  |  |  |
| ENSG00000099194 | SCD | 1.04 | 0.22 | 4.797 | 1.61e-06 | 6.40e-04 |  |  |  |  |  |  |  |
| ENSG00000107282 | APBA1 | -0.43 | 0.09 | -4.768 | 1.86e-06 | 7.20e-04 |  |  |  |  |  |  |  |
| ENSG00000154814 | OXNAD1 | -0.40 | 0.08 | -4.762 | 1.92e-06 | 7.25e-04 |  |  |  |  |  |  |  |
| ENSG00000132953 | XPO4 | -0.54 | 0.11 | -4.727 | 2.28e-06 | 7.82e-04 |  |  |  |  |  |  |  |
| ENSG00000123700 | KCNJ2 | 0.42 | 0.09 | 4.668 | 3.04e-06 | 9.50e-04 |  |  |  |  |  |  |  |
| ENSG00000133794 | ARNTL | 0.54 | 0.12 | 4.665 | 3.09e-06 | 9.50e-04 |  |  |  |  |  |  |  |
| ENSG00000164197 | RNF180 | -0.35 | 0.08 | -4.616 | 3.91e-06 | 0.001 |  |  |  |  |  |  |  |
| ENSG00000144668 | ITGA9 | 0.38 | 0.08 | 4.611 | 4.01e-06 | 0.001 |  |  |  |  |  |  |  |
| ENSG00000137804 | NUSAP1 | 0.37 | 0.08 | 4.601 | 4.20e-06 | 0.001 |  |  |  |  |  |  |  |
| ENSG00000143549 | TPM3 | -0.44 | 0.10 | -4.552 | 5.32e-06 | 0.001 |  |  |  |  |  |  |  |
| ENSG00000226306 | NPY6R | -0.52 | 0.11 | -4.548 | 5.41e-06 | 0.001 |  |  |  |  |  |  |  |
| ENSG00000116741 | RGS2 | 0.70 | 0.15 | 4.544 | 5.51e-06 | 0.001 |  |  |  |  |  |  |  |
| ENSG00000159884 | CCDC107 | 0.43 | 0.10 | 4.538 | 5.68e-06 | 0.001 |  |  |  |  |  |  |  |
| ENSG00000184588 | PDE4B | 0.45 | 0.10 | 4.521 | 6.16e-06 | 0.002 |  |  |  |  |  |  |  |
| ENSG00000134986 | NREP | -0.48 | 0.11 | -4.513 | 6.39e-06 | 0.002 |  |  |  |  |  |  |  |
| ENSG00000105612 | DNASE2 | 0.51 | 0.11 | 4.499 | 6.84e-06 | 0.002 |  |  |  |  |  |  |  |
| ENSG00000066382 | MPPED2 | -0.48 | 0.11 | -4.489 | 7.15e-06 | 0.002 |  |  |  |  |  |  |  |
| ENSG00000147010 | SH3KBP1 | -0.36 | 0.08 | -4.469 | 7.85e-06 | 0.002 |  |  |  |  |  |  |  |
| ENSG00000108342 | CSF3 | -1.16 | 0.26 | -4.407 | 1.05e-05 | 0.002 |  |  |  |  |  |  |  |
| ENSG00000138061 | CYP1B1 | 0.47 | 0.11 | 4.404 | 1.06e-05 | 0.002 |  |  |  |  |  |  |  |
| ENSG00000162493 | PDPN | 0.35 | 0.08 | 4.408 | 1.04e-05 | 0.002 |  |  |  |  |  |  |  |
| ENSG00000196526 | AFAP1 | 0.51 | 0.12 | 4.416 | 1.01e-05 | 0.002 |  |  |  |  |  |  |  |
| ENSG00000225613 | Not mapped^d^ | 1.14 | 0.26 | 4.407 | 1.05e-05 | 0.002 |  |  |  |  |  |  |  |
| ENSG00000249464 | LINC01091 | 0.67 | 0.15 | 4.398 | 1.09e-05 | 0.002 |  |  |  |  |  |  |  |
| ENSG00000139998 | RAB15 | 0.53 | 0.12 | 4.385 | 1.16e-05 | 0.002 |  |  |  |  |  |  |  |
| ENSG00000138688 | KIAA1109 | -0.36 | 0.08 | -4.362 | 1.29e-05 | 0.002 |  |  |  |  |  |  |  |
| ENSG00000174437 | ATP2A2 | -0.44 | 0.10 | -4.361 | 1.30e-05 | 0.002 |  |  |  |  |  |  |  |
| ENSG00000119771 | KLHL29 | 0.53 | 0.12 | 4.351 | 1.35e-05 | 0.002 |  |  |  |  |  |  |  |
| ENSG00000134569 | LRP4 | 0.41 | 0.09 | 4.350 | 1.36e-05 | 0.002 |  |  |  |  |  |  |  |
| ENSG00000182985 | CADM1 | -0.35 | 0.08 | -4.352 | 1.35e-05 | 0.002 |  |  |  |  |  |  |  |
| ENSG00000139209 | SLC38A4 | 0.49 | 0.11 | 4.344 | 1.40e-05 | 0.002 |  |  |  |  |  |  |  |
| ENSG00000079156 | OSBPL6 | 0.37 | 0.08 | 4.340 | 1.42e-05 | 0.002 |  |  |  |  |  |  |  |
| ENSG00000077150 | NFKB2 | 0.42 | 0.10 | 4.333 | 1.47e-05 | 0.002 |  |  |  |  |  |  |  |
| ENSG00000163071 | SPATA18 | 0.52 | 0.12 | 4.323 | 1.54e-05 | 0.003 |  |  |  |  |  |  |  |
| ENSG00000180209 | MYLPF | 0.44 | 0.10 | 4.315 | 1.59e-05 | 0.003 |  |  |  |  |  |  |  |
| ENSG00000108960 | MMD | 0.35 | 0.08 | 4.302 | 1.69e-05 | 0.003 |  |  |  |  |  |  |  |
| ENSG00000176909 | MAMSTR | 0.52 | 0.12 | 4.297 | 1.73e-05 | 0.003 |  |  |  |  |  |  |  |
| ENSG00000138759 | FRAS1 | -0.37 | 0.09 | -4.251 | 2.13e-05 | 0.003 |  |  |  |  |  |  |  |
| ENSG00000186047 | DLEU7 | 0.93 | 0.22 | 4.249 | 2.15e-05 | 0.003 |  |  |  |  |  |  |  |
|  | DLEU1-AS1 | 0.93 | 0.22 | 4.249 | 2.15e-05 | 0.003 |  |  |  |  |  |  |  |
| ENSG00000164649 | CDCA7L | -0.44 | 0.10 | -4.239 | 2.25e-05 | 0.003 |  |  |  |  |  |  |  |
| ENSG00000156265 | MAP3K7CL | 0.48 | 0.11 | 4.221 | 2.43e-05 | 0.003 |  |  |  |  |  |  |  |
| ENSG00000060656 | PTPRU | 0.52 | 0.12 | 4.214 | 2.51e-05 | 0.003 |  |  |  |  |  |  |  |
| ENSG00000162552 | WNT4 | 0.72 | 0.17 | 4.193 | 2.76e-05 | 0.004 |  |  |  |  |  |  |  |
| ENSG00000197442 | MAP3K5 | -0.40 | 0.09 | -4.190 | 2.78e-05 | 0.004 |  |  |  |  |  |  |  |
| ENSG00000223749 | Not mapped^d^ | 1.35 | 0.32 | 4.187 | 2.82e-05 | 0.004 |  |  |  |  |  |  |  |
| ENSG00000175567 | UCP2 | 0.44 | 0.11 | 4.154 | 3.27e-05 | 0.004 |  |  |  |  |  |  |  |
| ENSG00000087903 | RFX2 | 0.56 | 0.13 | 4.135 | 3.55e-05 | 0.004 |  |  |  |  |  |  |  |
| ENSG00000138411 | HECW2 | -0.50 | 0.12 | -4.134 | 3.57e-05 | 0.004 |  |  |  |  |  |  |  |
| ENSG00000233621 | LINC01137 | 0.67 | 0.16 | 4.137 | 3.52e-05 | 0.004 |  |  |  |  |  |  |  |
| ENSG00000260337 | Not mapped^d^ | 0.76 | 0.18 | 4.133 | 3.57e-05 | 0.004 |  |  |  |  |  |  |  |
| ENSG00000163823 | CCR1 | -0.65 | 0.16 | -4.127 | 3.67e-05 | 0.004 |  |  |  |  |  |  |  |
| ENSG00000106070 | GRB10 | -0.39 | 0.09 | -4.121 | 3.77e-05 | 0.004 |  |  |  |  |  |  |  |
| ENSG00000174791 | RIN1 | 0.96 | 0.23 | 4.108 | 3.99e-05 | 0.005 |  |  |  |  |  |  |  |
| ENSG00000196440 | ARMCX4 | 0.40 | 0.10 | 4.105 | 4.05e-05 | 0.005 |  |  |  |  |  |  |  |
| ENSG00000111602 | TIMELESS | 0.39 | 0.10 | 4.100 | 4.14e-05 | 0.005 |  |  |  |  |  |  |  |
| ENSG00000144908 | ALDH1L1 | 0.42 | 0.10 | 4.095 | 4.22e-05 | 0.005 |  |  |  |  |  |  |  |
| ENSG00000166833 | NAV2 | -0.40 | 0.10 | -4.093 | 4.25e-05 | 0.005 |  |  |  |  |  |  |  |
| ENSG00000101306 | MYLK2 | 0.35 | 0.09 | 4.079 | 4.51e-05 | 0.005 |  |  |  |  |  |  |  |
| ENSG00000285820 | Not mapped^d^ | 1.43 | 0.35 | 4.076 | 4.58e-05 | 0.005 |  |  |  |  |  |  |  |
| ENSG00000129910 | CDH15 | 0.35 | 0.09 | 3.984 | 6.76e-05 | 0.007 |  |  |  |  |  |  |  |
| ENSG00000254901 | BORCS8 | 0.37 | 0.09 | 3.975 | 7.05e-05 | 0.007 |  |  |  |  |  |  |  |
| ENSG00000158486 | DNAH3 | -0.84 | 0.22 | -3.922 | 8.79e-05 | 0.008 |  |  |  |  |  |  |  |
| ENSG00000260391 | Not mapped^d^ | 1.47 | 0.37 | 3.921 | 8.82e-05 | 0.008 |  |  |  |  |  |  |  |
| ENSG00000105327 | BBC3 | 0.72 | 0.19 | 3.903 | 9.50e-05 | 0.009 |  |  |  |  |  |  |  |
| ENSG00000183010 | PYCR1 | 0.66 | 0.17 | 3.898 | 9.69e-05 | 0.009 |  |  |  |  |  |  |  |
| ENSG00000226833 | LOC100505774 | -0.51 | 0.13 | -3.892 | 9.93e-05 | 0.009 |  |  |  |  |  |  |  |
|  | LOC112267877 | -0.51 | 0.13 | -3.892 | 9.93e-05 | 0.009 |  |  |  |  |  |  |  |
| ENSG00000109061 | MYH1 | 0.68 | 0.18 | 3.889 | 1.01e-04 | 0.009 |  |  |  |  |  |  |  |
| ENSG00000089101 | CFAP61 | 0.52 | 0.13 | 3.878 | 1.05e-04 | 0.009 |  |  |  |  |  |  |  |
| ENSG00000168334 | XIRP1 | 0.42 | 0.11 | 3.857 | 1.15e-04 | 0.010 |  |  |  |  |  |  |  |
| ENSG00000178752 | ERFE | 0.83 | 0.21 | 3.851 | 1.17e-04 | 0.010 |  |  |  |  |  |  |  |
| ENSG00000272734 | Not mapped^d^ | 0.43 | 0.11 | 3.853 | 1.17e-04 | 0.010 |  |  |  |  |  |  |  |
| ENSG00000105339 | DENND3 | -0.35 | 0.09 | -3.847 | 1.20e-04 | 0.010 |  |  |  |  |  |  |  |
| ENSG00000115129 | TP53I3 | 0.65 | 0.17 | 3.837 | 1.24e-04 | 0.010 |  |  |  |  |  |  |  |
| ENSG00000169710 | FASN | 0.78 | 0.20 | 3.838 | 1.24e-04 | 0.010 |  |  |  |  |  |  |  |
| ENSG00000169515 | CCDC8 | 0.72 | 0.19 | 3.827 | 1.30e-04 | 0.010 |  |  |  |  |  |  |  |
| ENSG00000176749 | CDK5R1 | 0.40 | 0.11 | 3.818 | 1.35e-04 | 0.010 |  |  |  |  |  |  |  |
| ENSG00000109771 | LRP2BP | 0.44 | 0.12 | 3.812 | 1.38e-04 | 0.011 |  |  |  |  |  |  |  |
| ENSG00000068724 | TTC7A | 0.43 | 0.11 | 3.809 | 1.40e-04 | 0.011 |  |  |  |  |  |  |  |
| ENSG00000138615 | CILP | 0.40 | 0.11 | 3.806 | 1.41e-04 | 0.011 |  |  |  |  |  |  |  |
| ENSG00000109321 | AREG | 1.12 | 0.30 | 3.799 | 1.46e-04 | 0.011 |  |  |  |  |  |  |  |
| ENSG00000157330 | C1orf158 | 1.58 | 0.42 | 3.793 | 1.49e-04 | 0.011 |  |  |  |  |  |  |  |
| ENSG00000196296 | ATP2A1 | 0.43 | 0.11 | 3.796 | 1.47e-04 | 0.011 |  |  |  |  |  |  |  |
| ENSG00000228526 | MIR34AHG | 0.48 | 0.13 | 3.792 | 1.49e-04 | 0.011 |  |  |  |  |  |  |  |
| ENSG00000161513 | FDXR | 0.62 | 0.16 | 3.784 | 1.54e-04 | 0.011 |  |  |  |  |  |  |  |
| ENSG00000174032 | SLC25A30 | -0.39 | 0.10 | -3.775 | 1.60e-04 | 0.011 |  |  |  |  |  |  |  |
| ENSG00000104147 | OIP5 | 0.50 | 0.13 | 3.773 | 1.61e-04 | 0.011 |  |  |  |  |  |  |  |
| ENSG00000205106 | LINC02716 | 0.59 | 0.16 | 3.772 | 1.62e-04 | 0.011 |  |  |  |  |  |  |  |
| ENSG00000099999 | RNF215 | 0.42 | 0.11 | 3.760 | 1.70e-04 | 0.012 |  |  |  |  |  |  |  |
| ENSG00000196482 | ESRRG | -0.38 | 0.10 | -3.731 | 1.91e-04 | 0.013 |  |  |  |  |  |  |  |
| ENSG00000267080 | ASB16-AS1 | 0.36 | 0.10 | 3.713 | 2.05e-04 | 0.014 |  |  |  |  |  |  |  |
| ENSG00000205959 | Not mapped^d^ | 0.39 | 0.11 | 3.684 | 2.30e-04 | 0.015 |  |  |  |  |  |  |  |
| ENSG00000138835 | RGS3 | -0.53 | 0.14 | -3.674 | 2.39e-04 | 0.015 |  |  |  |  |  |  |  |
| ENSG00000184545 | DUSP8 | 0.46 | 0.12 | 3.674 | 2.39e-04 | 0.015 |  |  |  |  |  |  |  |
| ENSG00000137193 | PIM1 | 0.46 | 0.13 | 3.669 | 2.43e-04 | 0.015 |  |  |  |  |  |  |  |
| ENSG00000262468 | Not mapped^d^ | 0.51 | 0.14 | 3.665 | 2.47e-04 | 0.015 |  |  |  |  |  |  |  |
| ENSG00000023171 | GRAMD1B | 0.44 | 0.12 | 3.661 | 2.51e-04 | 0.015 |  |  |  |  |  |  |  |
| ENSG00000146166 | LGSN | -1.09 | 0.30 | -3.658 | 2.54e-04 | 0.015 |  |  |  |  |  |  |  |
| ENSG00000147256 | ARHGAP36 | 0.78 | 0.21 | 3.652 | 2.60e-04 | 0.016 |  |  |  |  |  |  |  |
| ENSG00000159259 | CHAF1B | 0.36 | 0.10 | 3.653 | 2.59e-04 | 0.016 |  |  |  |  |  |  |  |
| ENSG00000124587 | PEX6 | 0.44 | 0.12 | 3.634 | 2.79e-04 | 0.016 |  |  |  |  |  |  |  |
| ENSG00000215018 | COL28A1 | 0.35 | 0.10 | 3.607 | 3.10e-04 | 0.017 |  |  |  |  |  |  |  |
| ENSG00000139292 | LGR5 | -0.49 | 0.14 | -3.595 | 3.25e-04 | 0.018 |  |  |  |  |  |  |  |
| ENSG00000099308 | MAST3 | 0.66 | 0.18 | 3.589 | 3.32e-04 | 0.018 |  |  |  |  |  |  |  |
| ENSG00000102468 | HTR2A | -0.81 | 0.23 | -3.589 | 3.32e-04 | 0.018 |  |  |  |  |  |  |  |
| ENSG00000110660 | SLC35F2 | 0.54 | 0.15 | 3.586 | 3.36e-04 | 0.018 |  |  |  |  |  |  |  |
| ENSG00000089847 | ANKRD24 | 0.70 | 0.19 | 3.583 | 3.40e-04 | 0.018 |  |  |  |  |  |  |  |
| ENSG00000118515 | SGK1 | 0.44 | 0.12 | 3.583 | 3.40e-04 | 0.018 |  |  |  |  |  |  |  |
| ENSG00000124935 | SCGB1D2 | -0.74 | 0.21 | -3.556 | 3.76e-04 | 0.020 |  |  |  |  |  |  |  |
| ENSG00000163492 | CCDC141 | -0.44 | 0.12 | -3.553 | 3.81e-04 | 0.020 |  |  |  |  |  |  |  |
| ENSG00000184349 | EFNA5 | 0.60 | 0.17 | 3.551 | 3.84e-04 | 0.020 |  |  |  |  |  |  |  |
| ENSG00000064655 | EYA2 | 0.60 | 0.17 | 3.541 | 3.99e-04 | 0.020 |  |  |  |  |  |  |  |
| ENSG00000091513 | TF | 0.43 | 0.12 | 3.540 | 4.00e-04 | 0.020 |  |  |  |  |  |  |  |
| ENSG00000138379 | MSTN | 0.47 | 0.13 | 3.544 | 3.94e-04 | 0.020 |  |  |  |  |  |  |  |
| ENSG00000184347 | SLIT3 | 0.36 | 0.10 | 3.533 | 4.11e-04 | 0.020 |  |  |  |  |  |  |  |
| ENSG00000235070 | Not mapped^d^ | -0.59 | 0.17 | -3.528 | 4.19e-04 | 0.021 |  |  |  |  |  |  |  |
| ENSG00000163879 | DNALI1 | 0.39 | 0.11 | 3.518 | 4.36e-04 | 0.021 |  |  |  |  |  |  |  |
| ENSG00000119969 | HELLS | 0.53 | 0.15 | 3.505 | 4.57e-04 | 0.022 |  |  |  |  |  |  |  |
| ENSG00000175489 | LRRC25 | -0.57 | 0.16 | -3.495 | 4.74e-04 | 0.022 |  |  |  |  |  |  |  |
| ENSG00000185105 | MYADML2 | 0.36 | 0.10 | 3.492 | 4.79e-04 | 0.023 |  |  |  |  |  |  |  |
| ENSG00000104313 | EYA1 | -0.42 | 0.12 | -3.489 | 4.85e-04 | 0.023 |  |  |  |  |  |  |  |
| ENSG00000258647 | Not mapped^d^ | 0.74 | 0.21 | 3.483 | 4.96e-04 | 0.023 |  |  |  |  |  |  |  |
| ENSG00000260604 | Not mapped^d^ | -0.63 | 0.18 | -3.484 | 4.94e-04 | 0.023 |  |  |  |  |  |  |  |
| ENSG00000278464 | Not mapped^d^ | 0.43 | 0.12 | 3.483 | 4.95e-04 | 0.023 |  |  |  |  |  |  |  |
| ENSG00000075240 | GRAMD4 | 0.37 | 0.11 | 3.472 | 5.16e-04 | 0.023 |  |  |  |  |  |  |  |
| ENSG00000086967 | MYBPC2 | 0.41 | 0.12 | 3.473 | 5.15e-04 | 0.023 |  |  |  |  |  |  |  |
| ENSG00000145626 | UGT3A1 | 0.41 | 0.12 | 3.477 | 5.07e-04 | 0.023 |  |  |  |  |  |  |  |
| ENSG00000161036 | LRWD1 | 0.51 | 0.15 | 3.471 | 5.19e-04 | 0.023 |  |  |  |  |  |  |  |
| ENSG00000212907 | ND4L | -0.35 | 0.10 | -3.464 | 5.31e-04 | 0.023 |  |  |  |  |  |  |  |
| ENSG00000198915 | RASGEF1A | -0.62 | 0.18 | -3.459 | 5.41e-04 | 0.023 |  |  |  |  |  |  |  |
| ENSG00000106992 | AK1 | 0.40 | 0.12 | 3.454 | 5.53e-04 | 0.024 |  |  |  |  |  |  |  |
| ENSG00000277758 | LOC102724488 | 0.89 | 0.26 | 3.432 | 6.00e-04 | 0.025 |  |  |  |  |  |  |  |
| ENSG00000197361 | FBXL22 | 0.49 | 0.14 | 3.404 | 6.63e-04 | 0.027 |  |  |  |  |  |  |  |
| ENSG00000231607 | DLEU2 | 0.35 | 0.10 | 3.393 | 6.90e-04 | 0.028 |  |  |  |  |  |  |  |
| ENSG00000158008 | EXTL1 | -0.58 | 0.17 | -3.392 | 6.94e-04 | 0.028 |  |  |  |  |  |  |  |
| ENSG00000140798 | ABCC12 | -0.70 | 0.21 | -3.389 | 7.01e-04 | 0.028 |  |  |  |  |  |  |  |
| ENSG00000165887 | ANKRD2 | 0.69 | 0.21 | 3.383 | 7.16e-04 | 0.028 |  |  |  |  |  |  |  |
| ENSG00000105877 | DNAH11 | 0.98 | 0.29 | 3.383 | 7.18e-04 | 0.028 |  |  |  |  |  |  |  |
| ENSG00000156463 | SH3RF2 | 0.40 | 0.12 | 3.373 | 7.45e-04 | 0.028 |  |  |  |  |  |  |  |
| ENSG00000285155 | Not mapped^d^ | -0.39 | 0.11 | -3.372 | 7.45e-04 | 0.028 |  |  |  |  |  |  |  |
| ENSG00000168528 | SERINC2 | 0.51 | 0.15 | 3.366 | 7.62e-04 | 0.029 |  |  |  |  |  |  |  |
| ENSG00000188488 | SERPINA5 | -0.69 | 0.21 | -3.356 | 7.90e-04 | 0.030 |  |  |  |  |  |  |  |
| ENSG00000125844 | RRBP1 | 0.36 | 0.11 | 3.348 | 8.13e-04 | 0.030 |  |  |  |  |  |  |  |
| ENSG00000108932 | SLC16A6 | 0.58 | 0.18 | 3.336 | 8.51e-04 | 0.031 |  |  |  |  |  |  |  |
| ENSG00000130600 | H19 | 0.56 | 0.17 | 3.330 | 8.67e-04 | 0.031 |  |  |  |  |  |  |  |
| ENSG00000154080 | CHST9 | -0.56 | 0.17 | -3.336 | 8.49e-04 | 0.031 |  |  |  |  |  |  |  |
| ENSG00000174996 | KLC2 | 0.39 | 0.12 | 3.331 | 8.66e-04 | 0.031 |  |  |  |  |  |  |  |
| ENSG00000188582 | PAQR9 | -0.47 | 0.14 | -3.337 | 8.48e-04 | 0.031 |  |  |  |  |  |  |  |
| ENSG00000284820 | Not mapped^d^ | 0.61 | 0.18 | 3.331 | 8.65e-04 | 0.031 |  |  |  |  |  |  |  |
| ENSG00000171617 | ENC1 | 0.41 | 0.12 | 3.328 | 8.74e-04 | 0.031 |  |  |  |  |  |  |  |
| ENSG00000047662 | FAM184B | 0.72 | 0.22 | 3.316 | 9.12e-04 | 0.032 |  |  |  |  |  |  |  |
| ENSG00000172932 | ANKRD13D | 0.41 | 0.13 | 3.314 | 9.21e-04 | 0.032 |  |  |  |  |  |  |  |
| ENSG00000158458 | NRG2 | 0.63 | 0.19 | 3.313 | 9.24e-04 | 0.032 |  |  |  |  |  |  |  |
| ENSG00000279529 | Not mapped^d^ | 0.43 | 0.13 | 3.309 | 9.37e-04 | 0.032 |  |  |  |  |  |  |  |
| ENSG00000284693 | LINC02606 | -0.48 | 0.14 | -3.311 | 9.29e-04 | 0.032 |  |  |  |  |  |  |  |
| ENSG00000140795 | MYLK3 | -0.35 | 0.11 | -3.303 | 9.57e-04 | 0.033 |  |  |  |  |  |  |  |
| ENSG00000146005 | PSD2 | 0.79 | 0.24 | 3.301 | 9.63e-04 | 0.033 |  |  |  |  |  |  |  |
| ENSG00000148671 | ADIRF | 0.60 | 0.18 | 3.301 | 9.63e-04 | 0.033 |  |  |  |  |  |  |  |
| ENSG00000111245 | MYL2 | -0.35 | 0.11 | -3.281 | 0.001 | 0.034 |  |  |  |  |  |  |  |
| ENSG00000176134 | Not mapped^d^ | -0.42 | 0.13 | -3.274 | 0.001 | 0.035 |  |  |  |  |  |  |  |
| ENSG00000071564 | TCF3 | 0.38 | 0.12 | 3.272 | 0.001 | 0.035 |  |  |  |  |  |  |  |
| ENSG00000214942 | Not mapped^d^ | -0.72 | 0.22 | -3.270 | 0.001 | 0.035 |  |  |  |  |  |  |  |
| ENSG00000005206 | SPPL2B | 0.37 | 0.11 | 3.262 | 0.001 | 0.036 |  |  |  |  |  |  |  |
| ENSG00000181418 | DDN | 0.74 | 0.23 | 3.253 | 0.001 | 0.037 |  |  |  |  |  |  |  |
| ENSG00000215187 | FAM166B | 0.44 | 0.14 | 3.254 | 0.001 | 0.037 |  |  |  |  |  |  |  |
| ENSG00000052749 | RRP12 | 0.42 | 0.13 | 3.252 | 0.001 | 0.037 |  |  |  |  |  |  |  |
| ENSG00000264343 | NOTCH2NLA | 0.47 | 0.14 | 3.244 | 0.001 | 0.037 |  |  |  |  |  |  |  |
| ENSG00000173546 | CSPG4 | 0.46 | 0.14 | 3.231 | 0.001 | 0.038 |  |  |  |  |  |  |  |
| ENSG00000177551 | NHLH2 | 1.15 | 0.36 | 3.230 | 0.001 | 0.038 |  |  |  |  |  |  |  |
| ENSG00000117707 | PROX1 | -0.36 | 0.11 | -3.227 | 0.001 | 0.039 |  |  |  |  |  |  |  |
| ENSG00000225472 | Not mapped^d^ | -0.51 | 0.16 | -3.227 | 0.001 | 0.039 |  |  |  |  |  |  |  |
| ENSG00000159713 | TPPP3 | 0.55 | 0.17 | 3.226 | 0.001 | 0.039 |  |  |  |  |  |  |  |
| ENSG00000205279 | CTXN3 | -0.72 | 0.22 | -3.221 | 0.001 | 0.039 |  |  |  |  |  |  |  |
| ENSG00000255495 | Not mapped^d^ | 0.40 | 0.12 | 3.220 | 0.001 | 0.039 |  |  |  |  |  |  |  |
| ENSG00000149090 | PAMR1 | 0.46 | 0.14 | 3.211 | 0.001 | 0.040 |  |  |  |  |  |  |  |
| ENSG00000124374 | PAIP2B | -0.35 | 0.11 | -3.193 | 0.001 | 0.041 |  |  |  |  |  |  |  |
| ENSG00000072310 | SREBF1 | 0.48 | 0.15 | 3.188 | 0.001 | 0.042 |  |  |  |  |  |  |  |
| ENSG00000104889 | RNASEH2A | 0.47 | 0.15 | 3.184 | 0.001 | 0.042 |  |  |  |  |  |  |  |
| ENSG00000238083 | LRRC37A2 | 0.36 | 0.11 | 3.185 | 0.001 | 0.042 |  |  |  |  |  |  |  |
| ENSG00000270021 | Not mapped^d^ | 0.52 | 0.16 | 3.184 | 0.001 | 0.042 |  |  |  |  |  |  |  |
| ENSG00000185847 | LINC01405 | -0.41 | 0.13 | -3.177 | 0.001 | 0.043 |  |  |  |  |  |  |  |
| ENSG00000248587 | Not mapped^d^ | 0.36 | 0.11 | 3.173 | 0.002 | 0.043 |  |  |  |  |  |  |  |
| ENSG00000105738 | SIPA1L3 | 0.37 | 0.12 | 3.161 | 0.002 | 0.044 |  |  |  |  |  |  |  |
| ENSG00000273301 | Not mapped^d^ | -0.86 | 0.27 | -3.145 | 0.002 | 0.046 |  |  |  |  |  |  |  |
| ENSG00000077943 | ITGA8 | -0.41 | 0.13 | -3.136 | 0.002 | 0.047 |  |  |  |  |  |  |  |
| ENSG00000241288 | LINC02614 | 0.43 | 0.14 | 3.138 | 0.002 | 0.047 |  |  |  |  |  |  |  |
| ENSG00000127191 | TRAF2 | 0.52 | 0.17 | 3.134 | 0.002 | 0.047 |  |  |  |  |  |  |  |
| ENSG00000283563 | ZCWPW2 | -0.36 | 0.11 | -3.129 | 0.002 | 0.047 |  |  |  |  |  |  |  |
| ENSG00000140280 | LYSMD2 | 0.37 | 0.12 | 3.127 | 0.002 | 0.047 |  |  |  |  |  |  |  |
| ENSG00000070601 | FRMPD1 | -0.36 | 0.12 | -3.117 | 0.002 | 0.048 |  |  |  |  |  |  |  |
| ENSG00000108231 | LGI1 | -0.37 | 0.12 | -3.112 | 0.002 | 0.049 |  |  |  |  |  |  |  |
| ENSG00000220563 | Not mapped^d^ | 0.37 | 0.12 | 3.110 | 0.002 | 0.049 |  |  |  |  |  |  |  |
| ENSG00000250303 | LINC02762 | -0.37 | 0.12 | -3.109 | 0.002 | 0.049 |  |  |  |  |  |  |  |
| ENSG00000166123 | GPT2 | -0.37 | 0.12 | -3.107 | 0.002 | 0.049 |  |  |  |  |  |  |  |
| ENSG00000167037 | SGSM1 | -0.60 | 0.19 | -3.102 | 0.002 | 0.049 |  |  |  |  |  |  |  |
| ENSG00000153822 | KCNJ16 | 0.59 | 0.19 | 3.098 | 0.002 | 0.049 |  |  |  |  |  |  |  |
| ^a^ *P*-values are adjusted for FDR. ^b^ Raw *P*-values from simulation based tests of uniformity of residuals where low values indicate problematic models. ^d^ No official gene symbol available, not included in enrichment analyses. | | | | | |  | |  |  |  |  |  |  |

**Supplementary Table 3.** Gene ontology (GO) analysis of genome-wide transcriptome data (RNA-seq; COPD vs. Healthy), performed as previously described.^E1,4^

| **Comparison** | **Gene set category** | **Gene set** | **Significance category^a^** | **Set size^b^** | **Rank *P*-value^c^** | **% MSD > 0^d^** | **GSEA *P*-value^e^** | **NES** | **LE^f^** | **Log_2_ Fold-change in LE [min, max]** |
| --- | --- | --- | --- | --- | --- | --- | --- | --- | --- | --- |
| Baseline: COPD vs. Healthy | Biological process | Actin filament based movement | Rank | 118 (153) | 4.47e-05 | 30.5% | 0.760 | 1.08 | 20 (85%) | 0.6 [0.29, 0.97] |
|  |  | Actin mediated cell contraction | Rank | 92 (123) | 4.29e-05 | 34.8% | 0.728 | 1.12 | 14 (92.9%) | 0.63 [0.41, 0.97] |
|  |  | Fatty acid metabolic process | Rank | 279 (396) | 8.48e-06 | 29.7% | 0.602 | -1.12 | 61 (83.6%) | -0.32 [-0.96, -0.15] |
|  |  | Monocarboxylic acid metabolic process | Rank | 469 (672) | 8.48e-06 | 29.4% | 0.468 | -1.17 | 72 (97.2%) | -0.36 [-1.17, -0.16] |
|  |  | Muscle contraction | Rank | 252 (362) | 4.27e-05 | 29.4% | 0.767 | 1.05 | 34 (82.4%) | 0.59 [0.25, 1.01] |
|  |  | Muscle filament sliding | Rank | 31 (39) | 1.39e-04 | 54.8% | 0.728 | 1.15 | 10 (90%) | 0.61 [0.29, 0.97] |
|  |  | Muscle system process | Rank | 321 (467) | 8.48e-06 | 29.9% | 0.740 | 1.08 | 45 (82.2%) | 0.56 [0.25, 1.03] |
|  | Cellular component | Inner mitochondrial membrane protein complex | GSEA | 114 (138) | 0.771 | 27.2% | 0.003 | -1.83 | 39 (76.9%) | -0.22 [-0.37, -0.12] |
|  |  | Mitochondrial matrix | GSEA | 436 (473) | 0.122 | 29.4% | 2.19e-04 | -1.61 | 120 (88.3%) | -0.23 [-0.53, -0.12] |
|  |  | Mitochondrial protein complex | GSEA | 234 (265) | 0.933 | 26.1% | 3.66e-05 | -1.90 | 70 (80%) | -0.21 [-0.37, -0.1] |
|  |  | Organelle inner membrane | GSEA | 461 (549) | 0.826 | 25.4% | 0.005 | -1.43 | 92 (95.7%) | -0.24 [-0.56, -0.14] |
|  |  | Actin cytoskeleton | Rank | 392 (503) | 1.87e-04 | 28.1% | 0.304 | 1.29 | 93 (74.2%) | 0.41 [0.14, 0.98] |
|  |  | Contractile fiber | Rank | 191 (238) | 2.24e-05 | 33% | 0.505 | 1.21 | 49 (83.7%) | 0.41 [0.16, 1] |
|  | Molecular function | G protein coupled receptor activity | GSEA | 146 (867) | 0.411 | 22.6% | 0.018 | -1.75 | 29 (69%) | -0.52 [-1.39, -0.17] |
| Post-RT (13 weeks training): ΔCOPD vs ΔHealthy | Biological process | Proteasomal protein catabolic process | Rank | 421 (481) | 0.019 | 29.2% | 0.591 | -1.08 | 97 (82.5%) | -0.43 [-1.26, -0.19] |
|  |  | Regulation of cholesterol efflux | Rank | 25 (46) | 0.019 | 48% | 0.102 | -1.50 | 13 (84.6%) | -0.54 [-1.32, -0.29] |
|  |  | Regulation of protein catabolic process | Rank | 327 (395) | 0.019 | 31.2% | 0.293 | -1.17 | 71 (97.2%) | -0.46 [-1.26, -0.23] |
|  | Cellular component | Actin cytoskeleton | Consensus | 392 (503) | 0.002 | 29.1% | 5.68e-06 | -1.38 | 133 (75.2%) | -0.44 [-1.17, -0.16] |
|  |  | Actin filament bundle | Consensus | 67 (75) | 5.45e-04 | 38.8% | 0.016 | -1.47 | 31 (74.2%) | -0.48 [-1.17, -0.2] |
|  |  | Actomyosin | Consensus | 69 (78) | 4.62e-04 | 37.7% | 0.011 | -1.48 | 31 (74.2%) | -0.49 [-1.17, -0.2] |
|  |  | Contractile fiber | Consensus | 191 (238) | 1.04e-05 | 33.5% | 1.56e-04 | -1.44 | 63 (87.3%) | -0.47 [-1.17, -0.19] |
|  |  | I band | Consensus | 114 (140) | 4.71e-04 | 33.3% | 2.28e-04 | -1.52 | 40 (87.5%) | -0.48 [-1.17, -0.2] |
|  |  | Adherens junction | GSEA | 127 (166) | 0.244 | 27.6% | 0.005 | -1.42 | 44 (65.9%) | -0.47 [-1.17, -0.16] |
|  |  | Cell cell junction | GSEA | 344 (493) | 0.161 | 26.5% | 1.91e-04 | -1.34 | 116 (63.8%) | -0.43 [-1.17, -0.16] |
|  |  | Cell substrate junction | GSEA | 359 (423) | 0.305 | 27.9% | 0.003 | -1.31 | 112 (68.8%) | -0.43 [-0.96, -0.16] |
|  |  | Collagen containing extracellular matrix | GSEA | 214 (427) | 0.999 | 20.1% | 0.005 | -1.34 | 74 (51.4%) | -0.47 [-1.56, -0.19] |
|  |  | Extrinsic component of cytoplasmic side of plasma membrane | GSEA | 65 (99) | 0.305 | 24.6% | 0.003 | -1.56 | 15 (100%) | -0.53 [-0.89, -0.3] |
|  |  | Extrinsic component of plasma membrane | GSEA | 109 (172) | 0.458 | 22% | 0.005 | -1.45 | 25 (84%) | -0.51 [-0.89, -0.27] |
|  |  | Polymeric cytoskeletal fiber | GSEA | 437 (756) | 0.110 | 25.9% | 0.005 | -1.25 | 135 (71.1%) | -0.43 [-1.17, -0.17] |
|  |  | Heterochromatin | Rank | 63 (78) | 0.004 | 39.7% | 0.063 | -1.40 | 19 (94.7%) | -0.48 [-1.05, -0.17] |
|  | Molecular function | Actin binding | Consensus | 336 (437) | 0.001 | 30.7% | 3.17e-07 | -1.42 | 125 (75.2%) | -0.44 [-1.17, -0.16] |
|  |  | Actin filament binding | Consensus | 162 (206) | 0.002 | 32.7% | 0.001 | -1.43 | 65 (70.8%) | -0.46 [-1.13, -0.21] |
|  |  | Chromatin binding | Consensus | 448 (596) | 0.001 | 29.2% | 0.025 | -1.23 | 94 (92.6%) | -0.44 [-1.05, -0.17] |
|  |  | Molecular adaptor activity | Consensus | 252 (314) | 0.001 | 30.6% | 0.048 | -1.25 | 80 (70%) | -0.44 [-1.14, -0.16] |
|  |  | Cell adhesion molecule binding | GSEA | 407 (544) | 0.384 | 25.3% | 5.69e-04 | -1.31 | 120 (75.8%) | -0.45 [-1.56, -0.16] |
|  |  | Protein kinase activity | GSEA | 449 (563) | 0.353 | 25.6% | 9.88e-04 | -1.29 | 102 (81.4%) | -0.49 [-1.29, -0.22] |
|  |  | Protein serine threonine kinase activity | GSEA | 361 (434) | 0.167 | 27.1% | 0.004 | -1.28 | 83 (84.3%) | -0.48 [-1.26, -0.22] |
|  |  | Glutamate receptor binding | Rank | 31 (46) | 0.016 | 54.8% | 0.071 | -1.49 | 16 (100%) | -0.41 [-0.69, -0.16] |
|  |  | Nuclear receptor binding | Rank | 83 (101) | 0.016 | 37.3% | 0.812 | -1.03 | 21 (90.5%) | -0.43 [-1.05, -0.16] |
|  |  | Protein macromolecule adaptor activity | Rank | 200 (244) | 6.58e-04 | 33% | 0.070 | -1.28 | 71 (69%) | -0.44 [-1.14, -0.16] |
|  |  | Signaling adaptor activity | Rank | 54 (68) | 0.016 | 40.7% | 0.343 | -1.25 | 22 (77.3%) | -0.42 [-0.77, -0.18] |
|  |  | Signaling receptor complex adaptor activity | Rank | 32 (41) | 0.016 | 43.8% | 0.267 | -1.32 | 10 (100%) | -0.49 [-0.77, -0.27] |
|  |  | Structural constituent of muscle | Rank | 33 (43) | 0.016 | 42.4% | 0.073 | -1.44 | 13 (92.3%) | -0.49 [-0.97, -0.26] |
|  |  | Ubiquitin binding | Rank | 71 (76) | 0.018 | 38% | 0.145 | -1.37 | 24 (91.7%) | -0.42 [-0.88, -0.25] |

^a^ Consensus significance indicates agreement between directional (GSEA) and non-directional (Rank) hypothesis test of overrepresentation (see methods for details). ^b^ Indicates number of identified genes in the gene set and total number of genes in the gene set in parentheses. ^c^ Rank-based enrichment test, based on minimum significant difference (MSD), identifies gene sets that are overrepresented among top-ranked genes without a directional hypothesis. ^d^ Fraction of genes in gene set with unadjusted 95% CI not spanning zero, i.e. MSD > 0. ^e^ Gene-set enrichment analysis (GSEA) tests for overrepresentation among top and bottom genes based on Log_2_ fold differences or changes × -log_10_(*P*-values) in comparing differences at baseline or changes from baseline between COPD and Healthy. Positive normalized enrichment score (NES) indicate gene sets with higher expression in COPD than Healthy; negative NES indicate gene sets with lower expression at respective time-points. ^f^ Number of genes in leading edge (LE, genes that contributes to the enrichment score) with the fraction of leading edge genes with unadjusted 95% CI not spanning zero.
